# Allele‐Specific Regulation of PAXIP1‐AS1 by SMC3/CEBPB at rs112651172 in Psychiatric Disorders Drives Synaptic and Behavioral Dysfunctions in Mice

**DOI:** 10.1002/advs.202508259

**Published:** 2025-09-05

**Authors:** Chaoying Ni, He Chen, Qiaqi Chen, Yangyang Liao, Yunqian Wang, Linyan Ye, Xiaohui Wu, Hongyu Ni, Tingyun Jiang, Shufen Li, Qiong Yang, Hong Xue, Zhongju Wang, Feng Yi, Cunyou Zhao

**Affiliations:** ^1^ Guangdong Provincial People's Hospital (Guangdong Academy of Medical Sciences) Key Laboratory of Mental Health of the Ministry of Education Guangdong‐Hong Kong‐Macao Greater Bay Area Center for Brain Science and Brain‐Inspired Intelligence Guangdong‐Hong Kong Joint Laboratory for Psychiatric Disorders Guangdong Province Key Laboratory of Psychiatric Disorders Guangdong Basic Research Center of Excellence for Integrated Traditional and Western Medicine for Qingzhi Diseases and School of Basic Medical Sciences Southern Medical University Guangzhou Guangdong 510515 China; ^2^ Department of Medical Genetics Guangdong Technology and Engineering Research Center for Molecular Diagnostics of Human Genetic Diseases Guangdong Engineering and Technology Research Center for Genetic Testing and Experimental Education/Administration Center School of Basic Medical Sciences Southern Medical University Guangzhou Guangdong 510515 China; ^3^ Department of Neurobiology School of Basic Medical Sciences Southern Medical University Guangzhou Guangdong 510515 China; ^4^ The Third People's Hospital of Zhongshan Zhongshan Guangdong 528451 China; ^5^ Department of Psychiatry The Affiliated Brain Hospital of Guangzhou Medical University (Guangzhou Huiai Hospital) Guangzhou Guangdong 510370 China; ^6^ Division of Life Science The Hong Kong University of Science and Technology Clear Water Bay Hong Kong 000000 China; ^7^ Gaozhou People's Hospital Gaozhou Guangdong 525000 China

**Keywords:** ASE, bipolar disorder, CNTNAP3, lncRNA, monozygotic twin, schizophrenia, ZGPAT

## Abstract

Schizophrenia (SCZ) and bipolar disorder (BPD) are highly heritable psychiatric disorders with complex genetic and environmental underpinnings. Allele‐specific expression (ASE) has emerged as a critical mechanism linking noncoding genetic variants to disease risk through epigenetic and environmental modulation. Here, whole‐genome and transcriptome analyses of monozygotic twin pairs discordant for BPD or SCZ are performed, identifying that noncoding genetic variants drive differential ASE patterns of long noncoding RNAs (lncRNAs) in affected individuals compared to their unaffected co‐twins. The rs112651172 (C/G) is identified as a functional ASE variant regulating *PAXIP1‐AS1* expression via allele‐specific transcription factor binding: SMC3 binds the C allele, while CEBPB binds the G allele, resulting in G allele‐specific upregulation in patients. Eevated *PAXIP1‐AS1* expression is consistently observed in SCZ and BPD postmortem brain tissues from PsychENCODE or LIBD datasets. In mice, G allele overexpression in the prefrontal cortex induces anxiety‐ and depression‐like behaviors, social deficits, memory impairments, sensorimotor gating abnormalities, and reduced neuronal excitability. Mechanistically, *PAXIP1‐AS1* upregualtes *CNTNAP3* by sequestering the transcriptional repressor ZGPAT. Knockdown of *CNTNAP3* reversed the observed phenotypes. These findings establish rs112651172 as a regulatory variant linking noncoding genetic risk to psychiatric phenotypeshrough ASE‐driven lncRNA dysregulation, suggesting new therapeutic targets in SCZ and BPD.

## Graphical abstract

This study identifies a functional noncoding variant, rs112651172 (C/G), that drives allele‐specific expression of *PAXIP1‐AS1* in monozygotic twin pairs discordant for schizophrenia and bipolar disorder. The risk G allele enhances CEBPB binding, leading to lncRNA upregulation, *CNTNAP3* derepression, and synaptic and behavioral deficits in mice. These findings highlight ASE‐mediated lncRNA dysregulation as a novel mechanism and therapeutic targets in psychiatric pathogenesis.

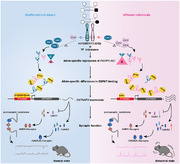

## 1 Introduction

Major psychiatric disorders, including schizophrenia (SCZ) and bipolar disorder (BPD), are highly heritable and complex mental illnesses.^[^ ^1^ ^]^ Recent studies have demonstrated a significant genetic overlap between these two disorders, with a high degree of genetic correlation^[^ ^2^ ^]^ and elevated relative risks among the relatives of both BPD and SCZ patients.^[^ ^3^, ^4^ ^]^ The complexity of these disorders arises not only from the heterogeneity of their phenotypes but also from their multi‐factorial nature, involving a combination of genetic, developmental, and environmental factors.^[^ ^5^, ^6^, ^7^ ^]^

In recent years, genome‐wide association studies (GWAS) have emerged as a powerful approach for identifying numerous genetic variants associated with SCZ or BPD. Notably, the majority of these variant are located in noncoding regions, suggesting that they may exert their effects by regulating gene expression rather than directly altering protein structure. Noncoding variants can influence gene function through mechanisms such as modulating transcriptional activity, RNA stability, transcription factor binding, or chromatin accessibility. A key mechanism underlying this regulatory effect is allele‐specific expression (ASE), a form of genetic regulation in which one allele exhibits preferential RNA expression at a specific locus.^[^ ^8^ ^]^ In an extreme example of ASE, genomic imprinting results in monoallelic expression, where a particular allele is entirely silenced. While ASE rarely leads to complete silencing of one allele, it reflects changes in the allelic expression ratio, mediated by cis‐regulatory elements, trans‐acting factors, and epigenetic modifications.^[^ ^9^, ^10^, ^11^ ^]^ The interplay of these complex regulatory mechanisms may contribute to the heterogeneity of allele expression patterns among individuals, often causing the same genetic variant to produce variable phenotypic manifestations. This variability in gene regulation and expression may underlie the phenotypic heterogeneity observed in complex psychiatric disorders, such as SCZ and BPD.

As essential epigenetic modifications, long noncoding RNAs (lncRNAs) are a class of RNA molecules longer than 200 nucleotides that lack protein‐coding potential. ≈40% of lncRNAs are specifically expressed in brain tissue, where they play critical roles in central nervous system development and contribute to psychiatric disorders susceptibility.^[^ ^12^, ^13^ ^]^ Most lncRNAs regulate gene expression at the transcriptional or post‐transcriptional level by interacting with the target DNA, RNAs, or proteins through base‐pairing or structural domains formation.^[^ ^14^, ^15^ ^]^ Similar to protein‐coding gene variations, genetic variations within lncRNA loci can alter their expression levels and functional properties, thereby influencing disease susceptibility.^[^ ^16^ ^]^ Additionally, ASE sites on noncoding RNAs may function as “gene dosage fine‐tuners,” modulating gene expression, epigenetic states, and molecular interaction networks. Dysregulated ASE patterns in lncRNAs may, in turn, disrupt psychiatric disease‐related pathways, contributing to the pathogenesis of disorders such as SCZ and BPD.

Here, we hypothesize that ASE of functional variants on lncRNAs contributes to the development of psychiatric disorders. To test this hypothesis, we analyzed ASE of lncRNAs by performing whole genome sequencing (WGS) and transcriptome sequencing (RNA‐seq) on monozygotic (MZ) twins discordant (DC) for SCZ and BPD. Through this analysis, we identified potential disease‐associated ASE event on lncRNAs and their regulatory trans‐acting factors. To validate the functional effects of these ASE variants, we conducted behavioral experiments and a mechanistic study in both mouse models and cellular systems. These experiments assessed how specific variants affect lncRNA function and explored the mechanisms underlying ASE transitions. Our findings suggest that ASE changes of functional variants on noncoding RNAs provide new insights into the genetic and epigenetic regulation of psychiatric diseases.

## 2 Results

### 2.1 Associations of Allele‐Specific LncRNA Expression Changes with Psychiatric Disorders

**Figure 1:**
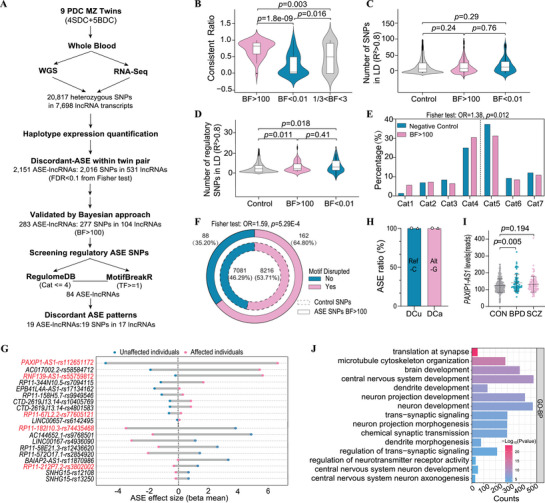
Identification and characterization of psychiatry‐related ASE sites. A) Flowchart illustrating the identification of psychiatry‐related ASE lncRNAs. B) Consistency analysis of disease‐related discordant ASE patterns across twin pairs, comparing ASE lncRNAs with BF > 100, BF < 0.01, or 1/3 < BF < 3. (C, D) Distribution of common variants C) or regulatory variants D) in linkage disequilibrium (LD; R^2^>0.8) with ASE SNPs, comparing those with BF>100 to those with BF<0.01 and control variants. Two‐side *p* values were calculated using the Wilcoxon Signed‐Rank Test in panels B‐D. E,F) Enrichment analysis of ASE SNPs (BF>100), showing their distribution across RegulomeDB categories 1–8 (E) and motif disruption effects identified using MotifBreakR (F). P values were calculated using the Fisher exact test. G) Effect size of 19 discordant ASE‐lncRNAs in DC twin pairs. Each data point represents the beta mean value at the ASE site with the strongest evidence of allelic imbalance, where positive values indicate higher expression of the alternative allele relative to the reference allele, and negative values indicate the opposite. Values are shown separately for affected and unaffected individuals within DC twin pairs, highlighting discordant ASE patterns. LncRNAs labeled in red represent genes with BPD‐ or SCZ‐associated differential expression in postmortem brain tissues based on the PsychENCODE dataset analyses. H) ASE switching patterns across twin pairs at rs112651172 (C/G), depicting the reference (Ref‐C) or alternative (Alt‐G) allelic level of *PAXIP1‐AS1* in unaffected (DCu) or affected (DCa) individuals from one SDC and one BDC twin pair. I) *PAXIP1‐AS1* expression level in the PFC of control (CON), bipolar disorder (BPD), and schizophrenia (SCZ) cohorts derived from the PsychENCODE dataset. P values were calculated using unpaired Student's *t*‐test. J) Gene Ontology‐Biological Process (GO‐BP) enrichment analysis of *PAXIP1‐AS1* co‐expressed genes in the GTEx brain dataset was performed using ToppGene Suite. The color scale indicates the log‐transformed uncorrected P values for each enriched term.

To investigate ASE related to psychiatric disorders, we collected peripheral blood for WGS and RNA‐seq from nine pairs of DC MZ twins (18 individuals), including four SCZ DC twins (SDC) and five BPD DC twins (BDC) (Table S1, Supporting Information). WGS genotyping identified 20,817 single nucleotide polymorphisms (SNPs) with heterozygous calls in at least one twin pairs, spanning 7,698 lncRNA transcripts (**Figure** **1**A). Using these genotypes, we constructed haplotypes and generated individual‐specific reference genome sequences. We then quantified haplotype‐level RNA expression and identified significant discordant ASE genes within each twin pair (Fisher test, FDR<0.1) by integrating the haplotype‐level expression with SNP genotypes (Table S2, Supporting Information). Across twin pairs, we identified 2,016 ASE SNPs in 531 lncRNA transcripts (resulting in 2,151 SNP‐isoform pairs, or ASE lncRNAs) exhibiting discordant ASE in at least one twin pair. We further filtered ASE SNPs to include only those detected in at least two twin pairs, yielding 726 ASE SNPs located 219 lncRNA transcripts (representing a total 754 ASE lncRNA events). These SNPs were then subjected to multi‐sample differential ASE analysis using Bayesian inference. To account for genetic background and haplotype differences, we integrated the 754 ASE lncRNAs using ASE SNPs as markers and performed a pooled Bayesian analysis of discordant ASE patterns across all twin pairs. Applying a stringent Bayesian factor threshold (BF>100), we identified 277 candidate ASE SNPs within 104 transcripts (representing 283 ASE lncRNA events) that showed strong phenotype‐specificity. To evaluate the consistency of disease‐related discordant ASE patterns across twin pairs, we performed a meta‐analysis and generated forest plots. ASE lncRNAs with BF>100 exhibited highly consistent ASE patterns (OR = 12.73, *p* = 1.958E‐20; Figure S1, Supporting Information), with significantly greater concordance among DC twin pairs compared to other ASE lncRNAs (Figure 1B).

To explore how ASE SNPs regulate their own expression, we analyzed their frequency distribution and linkage disequilibrium (LD) patterns. We found that the number of common variants (Figure 1C) or regulatory variants (Figure 1D) in strong LD (R^2^>0.8) with ASE SNPs showing strong phenotype‐specificity (BF >100) was not significantly higher than those associated with ASE SNPs lacking phenotype specificity (BF <0.01) or control variants. This observation suggests that ASE SNPs do not primarily regulate their own expression through linkage with other genetic variants. Notably, compared to control SNPs, both groups of ASE SNPs—those with strong phenotype specificity (BF >100) and those without phenotype specificity (BF < 0.01)—are linked to significantly more regulatory variants (Figure 1D). However, there is no significant difference between these two ASE groups. Therefore, the number of linked regulatory variants alone does not fully explain phenotype‐specific ASE. We propose that non‐genetic mechanisms, such as differential transcription factor (TF) expression or epigenetic modifications, likely play critical roles in driving disease‐specific ASE. We then conducted an enrichment analysis of ASE SNPs (BF >100) using RegulomeDB^[^ ^17^, ^18^ ^]^ and motifbreakR.^[^ ^19^, ^20^ ^]^ This analysis revealed a significant enrichment of these ASE SNPs in RegulomeDB categories 1–4 (OR = 1.38, *p* = 0.012; Figure 1E; Table S3, Supporting Information), suggesting their preference for regions near TF binding sites and open chromatin. Further MotifBreakR analysis indicated that ASE SNPs (BF >100) were more likely than control SNPs to affect TF binding (OR = 1.59, *p* = 5.29E‐4; Figure 1F; Table S4, Supporting Information). These findings suggest that ASE‐SNPs are located within open chromatin regions, where genetic variations may contribute to ASE by influencing TF binding.

### 2.2 Allelic Effects of rs112651172 on PAXIP1‐AS1 Expression in Psychiatric Disorders

We employed RegulomeDB and MotifbreakR tools to identify regulatory ASE SNPs among those with BF >100, resulting in the discovery of 84 ASE‐lncRNA transcripts (Figure 1A). These ASE‐lncRNAs were filtered based on their ASE effect size (beta mean), indicating ASE significance (Fisher test, *p* <0.01) in either unaffected or affected individuals. We identified 19 ASE SNPs corresponding to 17 lncRNA transcripts (19 ASE lncRNAs) that exhibited discordant ASE patterns in DC twin pairs, where the ASE effect size in unaffected individuals was opposite to that in affected individuals (Figure 1G; Table S5, Supporting Information). Among the 17 lncRNA transcripts, five showed differential expression in postmortem brain samples from individuals with SCZ or BPD, as observed in the PsychENCODE brain RNA‐seq dataset.^[^ ^21^ ^]^ Notably, the top‐ranked ASE SNP, rs112651172 (C/G, BF = 1.84E+06; Figure 1G), located on *PAXIP1‐AS1*, exhibits strong monoallelic expression. Specifically, unaffected individuals predominantly expressed the reference C allele, while affected individuals showed a pronounced shift toward expression of the alternative G allele across two DC twin pairs (one SDC and one BDC; Figure 1H). Furthermore, *PAXIP1‐AS1* expression was significantly increased in the prefrontal cortex (PFC) of individuals with BPD (Log2FC = 0.176, *p* = 0.0046; Figure 1I) and showed a similar trend in SCZ patients (Log2FC = 0.074, *p* = 0.1936) in the PsychENCODE BrainGVEX dataset.^[^ ^21^ ^]^ This effect was statistically significant in the dorsolateral prefrontal cortex (DLPFC) of SCZ patients (Log2FC = 0.0975, *p* = 3.2E‐5) in the LIBD dataset.^[^ ^22^ ^]^ Additionally, *PAXIP1‐AS1* expression was increased in the PFC of female individuals with major depressive disorder (MDD; Log2FC = 0.2615, *p* = 0.0396) and in the nucleus accumbens of male individuals with MDD (Log2FC = 0.2339, *p* = 0.0096).^[^ ^23^ ^]^

To explore the functional role of *PAXIP1‐AS1*, we performed a co‐expression analysis using the GTEx brain RNA‐seq dataset, identifying potential protein‐coding target genes associated with *PAXIP1‐AS1* expressions (Pearson |R|>0.7 and *p* <0.05). Gene ontology‐biological process (GO‐BP) analysis of these co‐expressed genes revealed significant enrichment in synaptic and central nervous system development processes, including translation at synapse, dendrite development, trans‐synaptic signaling, dendrite morphogenesis, central nervous system neuron development, and central nervous system neuron axonogenesis (Figure 1J). Given these findings, we investigated the allelic effects of rs112651172 (C/G) on *PAXIP1‐AS1* function and its potential role in psychiatric disorder pathogenesis.

### 2.3 Allele‐Specific Effects of PAXIP1‐AS1 Overexpression on Behavior and Cognition in Mice

The *PAXIP1‐AS1* transcript (also known as *PAXIP1‐DT*, ENSG00000273344.2) is located on the positive strand of chromosome 7 (chr7:155003448‐155005703), spanning 2,283 nucleotides with a single exon (Figure S2, Supporting Information). No homologous sequence exists in the mouse genome. RNA‐seq data from lncATLAS indicated that *PAXIP1‐AS1* is predominantly expressed in the nucleus (Figure S3, Supporting Information).^[^ ^24^ ^]^ To investigate the allele‐specific function of *PAXIP1‐AS1*‐rs112651172 (C/G), we generated two recombinant adeno‐associated viruses (rAAV). Each rAAV vector carried the *PAXIP1‐AS1* gene with either the reference C allele or the alternative G allele, driven by the human neuron‐specific synapsin I promoter. An empty AAV vector served as a control. Given the disease‐associated upregulations of *PAXIP1‐AS1* expression in the PFC, as observed in the PsychENCODE and LIBD datasets,^[^ ^21^, ^22^ ^]^ rAAV were injected into the PFC of male and female C57BL/6 wild‐type mice to mimic the overexpression state observed in patients (Figure S4A, Supporting Information). Behavioral assessments were conducted four weeks post‐rAAV administration (Figure S4B, Supporting Information).

**Figure 2:**
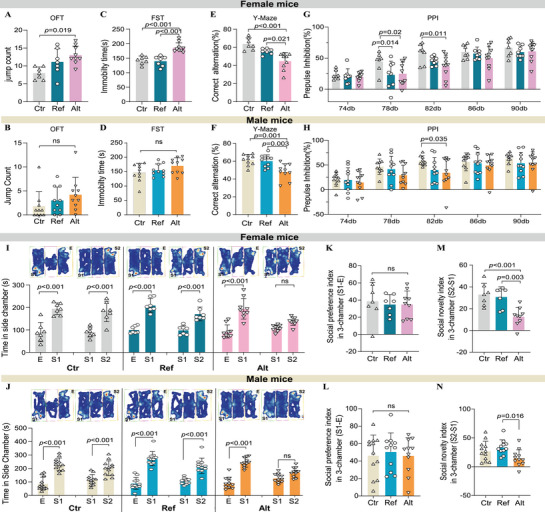
Allele‐specific behavioral and cognitive effects of PAXIP1‐AS1 overexpression in mice. A,B) Open field test (OFT) jump counts in Ctr (n = 7), Ref (n = 7) and Alt (n = 9) female mice (A) and Ctr (n = 10), Ref (n = 10) and Alt (n = 10) male mice (B), indicative of anxiety‐like behavior. C,D) Forced swimming test (FST) immobility time in Ctr (n = 7), Ref (n = 7) and Alt (n = 9) female mice (C) and Ctr (n = 10), Ref (n = 10) and Alt (n = 10) male mice (D), indicative of depression‐like behavior. E,F) Y‐maze spontaneous alternation in Ctr (n = 7), Ref (n = 7) and Alt (n = 9) female mice (E) and Ctr (n = 10), Ref (n = 10), and Alt (n = 10) male mice (F), measuring working memory performance. G,H) Prepulse inhibition (PPI) of acoustic startle response in Ctr (n = 7), Ref (n = 7) and Alt (n = 9) female mice (G) and Ctr (n = 9), Ref (n = 10) and Alt (n = 10) male mice (H), assessing sensorimotor gating. I–N) Three‐chamber social interaction test results in Ctr (n = 7), Ref (n = 7), and Alt (n = 9) female mice and Ctr (n = 12), Ref (n = 11), and Alt (n = 11) male mice. (I,J) Exploring times for empty cage (E) versus stranger mouse (S1), then a new stranger mouse (S2) for female (I) and male (J) mice. (K,L) Social preference index [(S1‐E)/total × 100% for female (K) and male (L) mice. (M,N) Social novelty index [(S2‐S1)/total × 100% for female (M) and male (N) mice. Data presented as mean ± SD for control (Ctr), PAXIP1‐AS1 reference allele (Ref), and PAXIP1‐AS1 alternative allele (Alt). Adjusted P values were calculated using ANOVA followed by Tukey's multiple comparison test for the indicated comparisons. *p* < 0.05 was considered statistically significant (ns, not significant).

In the open field test (OFT), female mice overexpressing the rs112651172 alternative G allele of *PAXIP1‐AS1* (Alt) exhibited significantly increased jump counts compared to the control (Ctr) group (**Figure** **2**A), with no differences observed in total travel distance or center exploration time (Figure S5A, Supporting Information). This suggests increased anxiety‐like behavior specifically in female Alt mice. Conversely, male Alt mice did not differ significantly in jump counts or center distances compared to Ref or Ctr groups (Figure 2B; Figure S5B, Supporting Information); however, they did show increased total distance traveled (Figure S5B, Supporting Information), indicating heightened general activity without clear anxiety‐related changes. In the forced swim test (FST), female Alt mice displayed significantly increased immobility time compared to Ref and Ctr mice (Figure 2C), indicating depressive‐like behavior; this effect was not observed in males (Figure 2D). In the Y‐maze, both male and female Alt mice exhibited significantly reduced spontaneous alternation, indicative of working memory deficits (Figure 2E,F; Figure S5C,D, Supporting Information). In the prepulse inhibition (PPI) test, both sexes showed significant reductions in PPI at 82 dB (Figure 2G,H). Additionally, female Alt mice exhibited impaired PPI at 78 dB, reflecting deficits in sensorimotor gating (Figure 2G).

In the three‐chamber social interaction test, all groups preferred the first stranger (S1) over an empty cage (Figure 2I,J), with no differences between groups (Figure 2K,L). However, when introduced to a second stranger (S2), female Alt mice exhibited significantly reduced preference for social novelty (S2 vs S1) compared to Ref and Ctr mice (Figure 2I,M). Male Alt mice also demonstrated reduced preference for S2 over S1 compared specifically to Ref mice (Figure 2J,N). These results indicated impaired social novelty recognition, with stronger effects in females. No significant differences were observed between groups of either sex in the elevated plus maze (EPM) or Barnes maze tests (Figure S5E–H, Supporting Information).

Collectively, these behavioral analyses demonstrate that *PAXIP1‐AS1* alternative G allele overexpression associates with anxiety‐ and depression‐like behaviors, short‐term memory deficits, impaired sensorimotor gating, and diminished social novelty recognition, with effects notably more pronounced in female mice.

### 2.4 Synaptic Dysfunction in PAXIP1‐AS1‐Alt Overexpressing Mice

**Figure 3:**
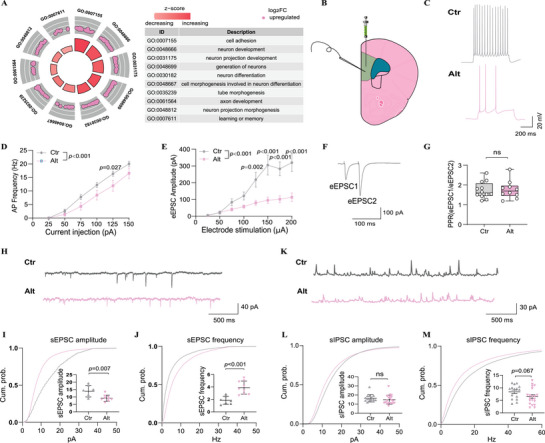
Electrophysiological alterations in PAXIP1‐AS1‐Alt overexpressing female mice. A) GO‐BP enrichment analysis of upregulated DEGs identified using DESeq2 with an ordinal categorical variable (Alt >Ref >Ctr). B) Schematic representation of whole‐cell patch‐clamp recordings performed on layer 5 pyramidal neurons of the PFC of mice. C,D) Representative action potential traces (C) and firing frequency (D) recorded from layer 5 cortical neurons of Ctr mice (20 neurons) and Alt mice (11 neurons). E–G) Amplitude of eEPSCs from Ctr mice (12 neurons) and Alt mice (10 neurons) (E) and representative traces of paired‐pulse stimulation responses (F) and PPRs of eEPSCs plotted against interstimulus intervals (G) recorded from Ctr mice (12 neurons) and Alt mice (10 neurons). H–M) Representative traces and cumulative probability plots of sEPSCs from Ctr mice (7 neurons) and Alt mice (9 neurons) (H–J) and sIPSC from Ctr mice (16 neurons) and Alt mice (20 neurons) (K–M) showing the amplitude and frequency differences recorded from the Ctr and Alt neurons. Data are presented as mean ± SD for cells obtained from at least three mice per group (Ctr or Alt). P values were calculated using two‐way ANOVA followed by Sidak's multiple comparisons test between Ctrl and OE mice in panels D and E. Unpaired two‐tailed Student's *t*‐tests were used for panels G, and I–M. *p* < 0.05 was considered statistically significant (ns, not significant).

To investigate the molecular basis of *PAXIP1‐AS1‐*induced behavioral abnormalities, we performed RNA‐seq analysis on the PFCs of Alt, Ref, and Ctr mice. Principal component analysis (PCA) and hierarchical clustering of gene expression data (FPKM values) revealed that Ref mice exhibited an intermediate expression profile between Alt and Ctr mice (Figure S6, Supporting Information). Differential gene expression analysis showed the highest number of differentially expressed genes (DEGs) in the Alt versus Ctr comparison, followed by Alt versus Ref and Ref versus Ctr. Using DESeq2^[^ ^25^ ^]^ with an ordinal categorical variable (Alt >Ref >Ctr), we identified 158 proteins encoding DEGs (|Log2FC|>0.5, FDR<0.05; Table S6, Supporting Information), including 85 upregulated and 73 downregulated genes. GO‐BP analysis revealed that upregulated DEGs were significantly enriched in cell adhesion, neuron development, neuron projection development, and generation of neurons (**Figure** **3**A). Conversely, downregulated DEGs were associated with mitochondrial respiratory chain complex assembly, protein‐containing complex assembly, mitochondrial electron transport, NADH to ubiquinone, and ATP synthesis coupled electron transport (Figure S7, Supporting Information). These findings, along with the enrichment of *PAXIP1‐AS1* co‐expressed genes in synaptic and central nervous system‐related functions in the human brain (Figure 1J), suggest that the behavioral abnormalities observed in *PAXIP1‐AS1*‐overexpressing mice may be linked to synaptic dysfunction.

To determine whether *PAXIP1‐AS1* overexpression results in alternative G allele‐specific synaptic alterations, we performed whole‐cell patch‐clamp recordings on layer 5 pyramidal neurons in acute PFC slice from Alt and Ctr female mice (Figure 3B). Intrinsic neuronal properties, including membrane resistance (Rm), membrane capacity (Cm), and resting membrane potential (RMP), did not differ significantly between groups (Figure S8, Supporting Information). However, Alt neurons exhibited reduced action potential firing in response to depolarizing current injections (Figure 3C,D), suggesting decreased neuronal excitability. Evoked excitatory postsynaptic currents (eEPSCs) were significantly attenuated in Alt mice compared to Ctr mice (Figure 3E), further indicating a reduction in excitatory synaptic transmission. To determine whether this deficit arises from presynaptic or postsynaptic mechanisms, we assessed the paired‐pulse ratio (PPR), an index of presynaptic neurotransmitter release probability. PPR values did not differ significantly between Alt and Ctr mice (Figure 3F,G), suggesting that the observed impairment is likely postsynaptic in origin. Supporting this interpretation, recordings of spontaneous excitatory postsynaptic currents (sEPSCs) revealed a significant reduction in sEPSCs amplitude in Alt neurons (Figure 3H,I), consistent with reduced postsynaptic receptor function. Interestingly, sEPSCs frequency was increased (Figure 3J), potentially reflecting a compensatory response to diminished postsynaptic. In addition, Alt neurons exhibited a similar amplitude and frequency of spontaneous inhibitory postsynaptic currents (sIPSCs) relative to controls (Figure 3K–M). Taken together, these results indicate that *PAXIP1‐AS1* influences neuronal activity in the PFC, with the alternative G allele specifically linked to reduced neuronal excitability and compromised excitatory synaptic transmission, primarily through postsynaptic mechanisms.

### 2.5 Modulation of CNTNAP3 Expression Restores Synaptic Function and Behavior in PAXIP1‐AS1‐Overexpressing Mice

**Figure 4:**
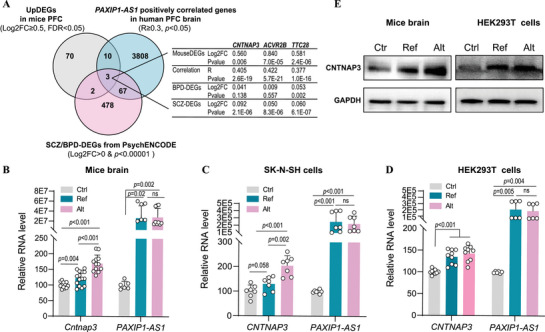
CNTNAP3 expression in *PAXIP1‐AS1‐*overexpressing mice and human cells. A) Venn diagram showing the overlap among upregulated DEGs in the PFC of Alt mice, *PAXIP1‐AS1* positively correlated genes in the human PFC, and SCZ‐ or BPD‐associated DEGs from the PsychENCODE dataset. B–D) qPCR analysis of *CNTNAP3* mRNA expression levels in PFC brain tissues of Ctr (n = 4), Ref (n = 5), and Alt (n = 4) mice (B), SH‐SY5Y cells (C), and HEK293T cells (D) from the same three groups. E) CNTNAP3 protein expression levels in mouse PFC tissues (**left**) and HEK293T cells (**right**) from Ctr, Ref, and Alt groups. Data are presented as mean ± SD. *P* values were calculated using an unpaired two‐tailed Student's t‐test for the indicated comparisons, with *p* < 0.05 considered statistically significant (ns, not significant).

To identify potential targets regulated by the *PAXIP1‐AS1* alternative G allele that contribute to synaptic dysfunction, we examined whether DEGs in mice were corresponding to human genes co‐expressed with *PAXIP1‐AS1* or associated with SCZ or BPD expression changes (**Figure** **4**A). Among 85 upregulated DEGs in the mouse PFC (Log2FC>0.5, FDR<0.05), three genes—*CNTNAP3*, *ACVR2B*, and *TTC28*—exhibited a positive expression correlation with *PAXIP1‐AS1* in GTEx human PFC brain RNA‐seq data and were upregulated in SCZ or BPD cases in the PsychENCODE brain RNA‐seq dataset.^[^ ^21^ ^]^ Of these, *CNTNAP3* (contactin‐associated protein‐like 3) is implicated in nervous system cell recognition and interacts with key synaptic adhesion molecules, including Neuroligin1, Neurogligin2 and the postsynaptic scaffolding proteins PSD95 and Gephyrin.^[^ ^26^ ^]^ It plays opposing roles in excitatory and inhibitory synapse development in vitro and in vivo^[^ ^26^ ^]^ and has been associated with SCZ, BPD and MDD.^[^ ^27^, ^28^, ^29^ ^]^ While *Cntnap3* knockout mice displayed typical motor function and anxiety levels, they exhibit mild impairments in motor learning and performance on the rota‐rod test.^[^ ^30^ ^]^ To validate these findings, we assessed *CNTNAP3* expression across three groups (Ctr, Ref and Alt mice). *CNTNAP3* mRNA levels increased progressively from Ctr to Ref to Alt in PFC tissues (Figure 4B), a pattern replicated in SK‐N‐SH (Figure 4C) and HEK293T (Figure 4D) cell lines via qPCR. CNTNAP3 protein levels followed the same trend in mouse PFC tissues and HEK293T cells (Figure 4E), suggesting that *CNTNAP3* is a key target of *PAXIP1‐AS1* alternative allele overexpression and may contribute to psychiatric‐disorder‐associated synaptic dysfunction.

**Figure 5:**
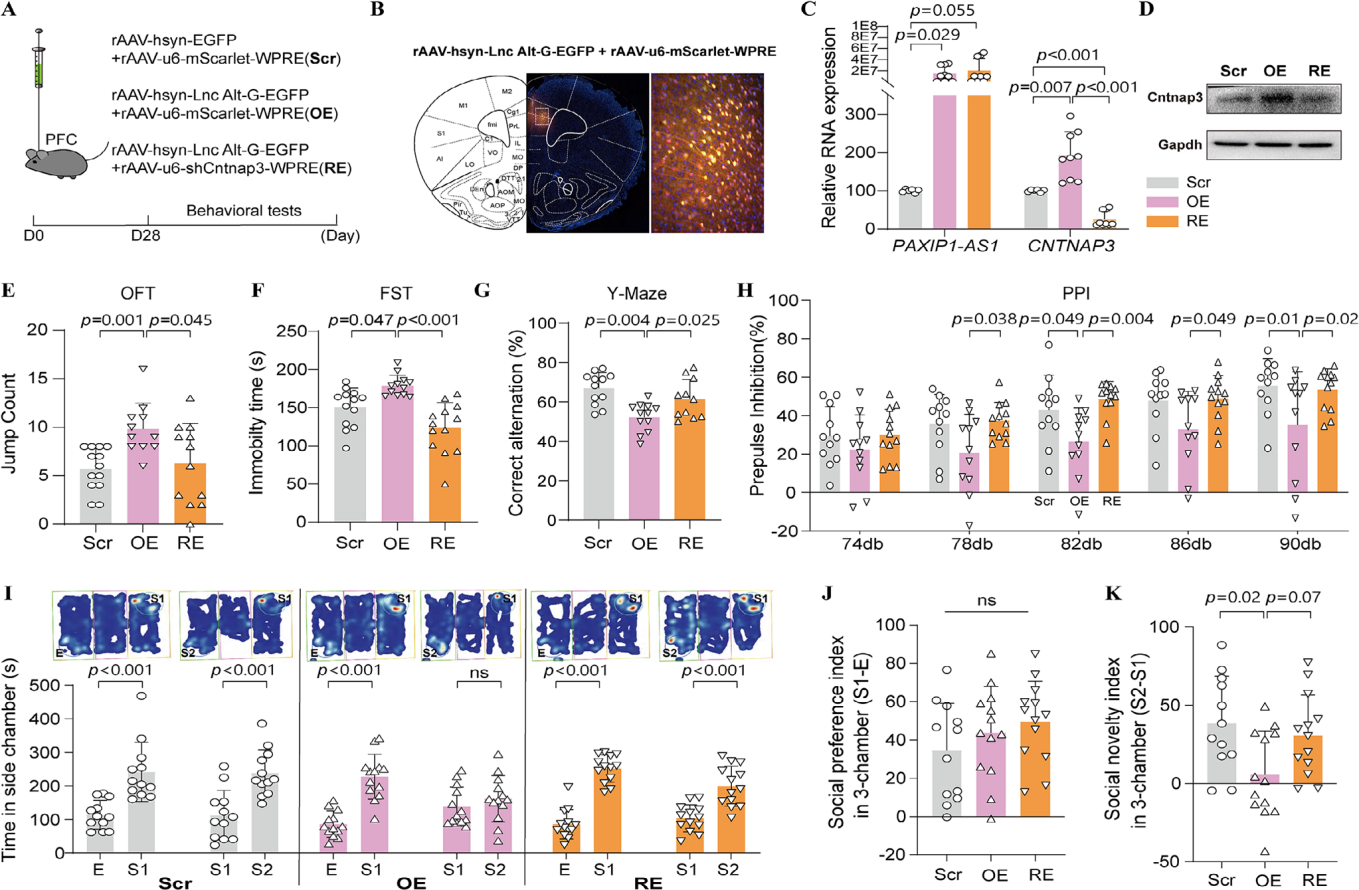
Modulation of Cntnap3 expression restores abnormal behaviors in PAXIP1‐AS1‐overexpressing female mice. A) Schematic representation of AAV injections into the PFC of C57BL/6 wild‐type mice. B) Fluorescence microscopy validation of viral expression in the PFC. C,D) qRT‐PCR (C) and Western blot (D) analysis confirming of Cntnap3 expression rescue in the PFC. Data from two Scr, three OE, and three RE mice E–H) Jump counts in the OFT from Scr (n = 13), OE (n = 11) and RE (n = 12) mice (E), immobility time in the FST from Scr (n = 13), OE (n = 12) and RE (n = 13) mice (F), spontaneous alternation counts in the Y‐maze test from Scr (n = 12), OE (n = 12) and RE (n = 11) mice (G) and PPI results from the acoustic startle response test in Scr (n = 11), OE (n = 11) and RE (n = 12) mice (H). I–K) Social interaction test in Scr (n = 12), OE (n = 13), and RE (n = 13) mice (I), as demonstrated with social preference index (J) and social novelty index (K). Data are presented as mean ± SD for Scr (gray bars), OE (pink bars), and RE (orange bars) groups, with at least eleven mice per group. Adjusted P values were calculated using ANOVA followed by Dunnett's T3 multiple comparisons test for panels C, E, and F, and Tukey's multiple comparison test for panels G‐K. p < 0.05 was considered statistically significant (ns, not significant).

To investigate whether reducing *Cntnap3* expression could mitigate behavioral and synaptic deficits in Alt mice, we injected rAAV carrying *Cntnap3*‐shRNA into the PFC of *PAXIP1‐AS1*‐Alt overexpressing (OE) mice (*Cntnap3*‐Rescue, RE; **Figure** **5**A). Viral injection sites were confirmed via fluorescence imaging (Figure 5B). qPCR and Western blot analysis verified significant *Cntnap3* knockdown at both mRNA (Figure 5C) and protein levels (Figure 5D), reversing the elevation observed in OE mice. Given that the behavioral effects of *PAXIP1‐AS1* overexpression were robust and statistically significant in female mice, we conducted subsequent behavioral testing in female mice used for the rescue experiment (RE group). The results showed that *Cntnap3* knockdown reduced anxiety‐ and depression‐like behaviors, as evidenced by decreased jump counts in the OFT (Figure 5E; Figure S9A, Supporting Information) and reduced immobility duration in the FST (Figure 5F). Additionally, *Cntnap3*‐RE mice exhibited improved short‐term memory (Figure 5G; Figure S9B, Supporting Information), sensorimotor gating (Figure 5H), and sociability (Figure 5I–K) compared to OE mice.

**Figure 6:**
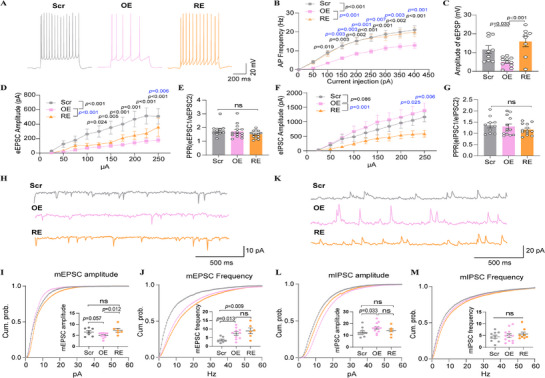
Modulation of CNTNAP3 expression restores synaptic dysfunction in PAXIP1‐AS1‐Overexpressing female mice. A,B) Representative action potential traces (A) and firing frequency (B) recorded from Scr mice (15 neurons), OE mice (17 neurons), and RE mice (14 neurons). C–E) Amplitude of eEPSPs from Scr mice (9 neurons), OE mice (11 neurons), and RE mice (8 neurons) (C) and eEPSCs from Scr mice (10 neurons), OE mice (14 neurons), and RE mice (12 neurons) (D), and PPRs of eEPSCs plotted against interstimulus intervals from Scr mice (10 neurons), OE mice (14 neurons), and RE mice (14 neurons) (E). F,G) Amplitude of eIPSCs from Scr mice (10 neurons), OE mice (13 neurons), and RE mice (12 neurons) (F) and PPRs of eIPSCs plotted against interstimulus intervals from Scr mice (10 neurons), OE mice (13 neurons), and RE mice (13 neurons) (G). H–M) Representative traces and cumulative probability plots of mEPSCs recorded from Scr mice (7 neurons), OE mice (10 neurons) and RE mice (6 neurons) (H–J) and mIPSC recorded from Scr mice (10 neurons), OE mice (12 neurons) and RE mice (8 neurons) (K–M), showing amplitude and frequency differences. Data are presented as mean ± SD for cells obtained from at least three mice per group (Scr, OE, and RE). Adjusted *p* values were calculated using ANOVA followed by Sidak's multiple comparisons test between Scr and OE mice (black *p* value), and OE and RE mice (blue *p* value) in panels B, D and F. Tukey's multiple comparisons test was used in panels C, E, and G. Unpaired two‐tailed Student's t‐tests were used in panels I–M. *p* < 0.05 was considered statistically significant (ns, not significant).

To assess synaptic function, whole‐cell patch‐clamp recordings of PFC neurons showed no significant differences in intrinsic neuronal properties, including membrane resistance (Rm), membrane capacity (Cm), and resting membrane potential (RMP), among the three groups (Figure S10A–C, Supporting Information). However, OE neurons exhibited reduced action potential firing upon the same depolarizing current injection, which was reversed in *Cntnap3*‐RE neurons (**Figure** **6**A,B). Similarly, both evoked excitatory postsynaptic potentials (eEPSPs; Figure 6C) and eEPSCs (Figure 6D) were diminished in OE neurons but restored in *Cntnap3*‐RE neurons, while PPR remained unchanged (Figure 6E; Figure S10D, Supporting Information), suggesting that the reduction in excitatory synaptic transmission was not due to altered presynaptic release probability. Conversely, evoked inhibitory postsynaptic currents (eIPSCs) were significantly elevated in OE neurons but normalized in *Cntnap3*‐RE neurons (Figure 6F), again with no change in PPR (Figure 6G; Figure S10E, Supporting Information), pointing to postsynaptic alterations. Further analysis of miniature excitatory (mEPSCs) and inhibitory (mIPSCs) postsynaptic currents in acute PFC slices, recorded in the presence of tetrodotoxin (TTX), revealed that mEPSC amplitude was mildly reduced in OE neurons but restored in *Cntnap3*‐RE neurons (Figure 6H,I). While mEPSC frequency was elevated in OE neurons (Figure 6J) and remained elevated following *Cntnap3* knockdown, suggesting a Cntnap3‐independent mechanism. Similarly, mIPSC amplitude was increased in OE neurons and was not reversed by *Cntnap3* knockdown (Figure 6K,L), while mIPSC frequency remained unchanged (Figure 6M). Together, these findings suggest that *PAXIP1‐AS1* overexpression disrupts excitatory/inhibitory (E/I) balance primarily through postsynaptic mechanisms, which partly mediated through CNTANP3, contributing to synaptic dysfunction.

**Figure 7:**
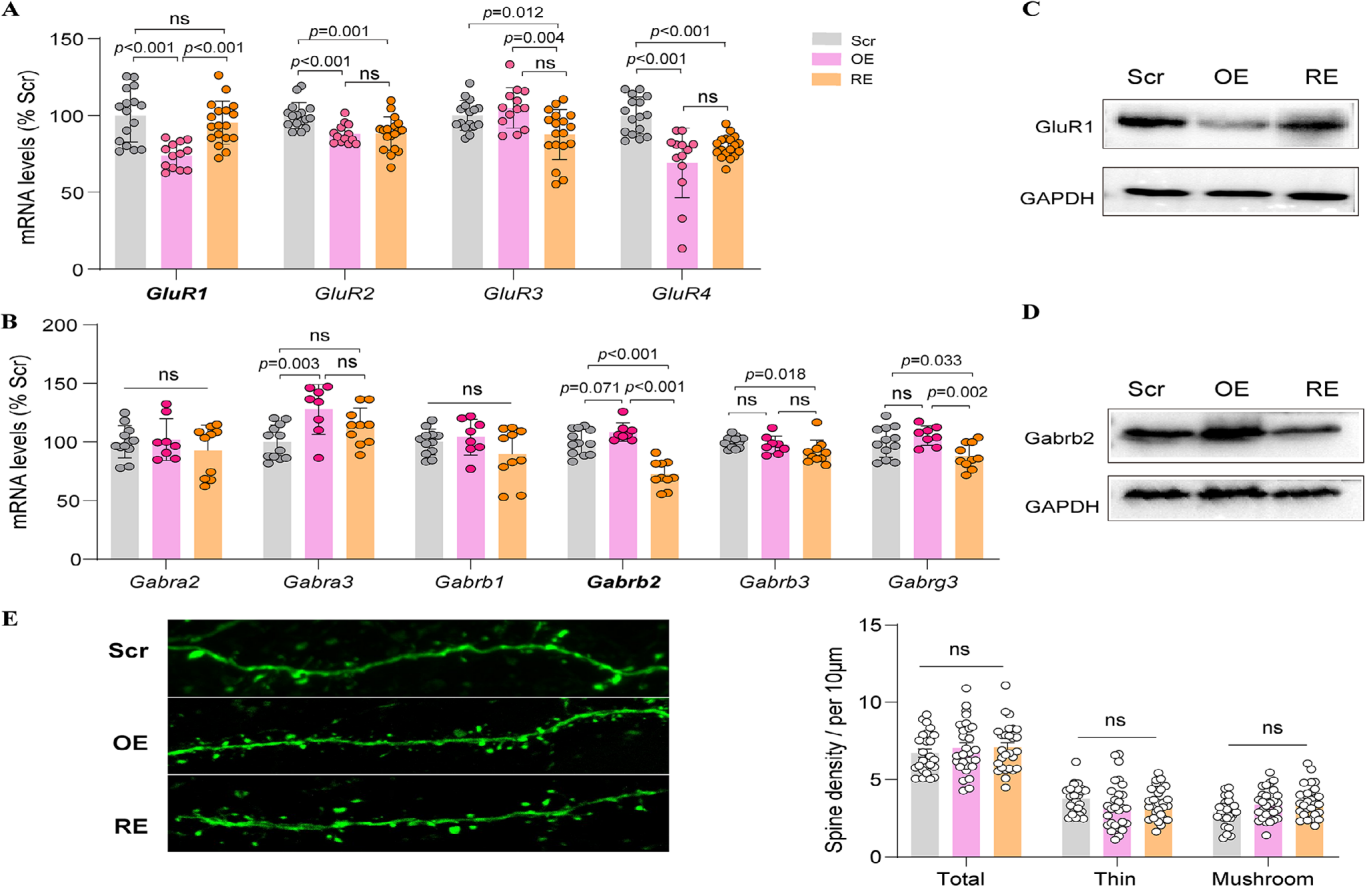
Expression of AMPA and GABA(A) receptor subunits and dendritic spine analysis in the PFC of mice. A,B) qPCR validation of AMPA receptor subunit from Scr (n = 6), OE (n = 6) and RE (n = 7) groups (A) and GABA(A) receptor subunit from Scr (n = 6), OE (n = 4) and RE (n = 5) groups (B) expressions in the PFC of mice. C,D) Western blot validation of GluR1 (C) and Gabrb2 (D) expression in the PFC of mice. E) Representative images of dendritic spine analysis in cortical layer 5 neurons of Scr (n = 3), OE (n = 3), and RE (n = 3) mice (left), showing no significant changes in spine density or morphology among the three groups (right). Data are presented as mean ± SD obtained from at least three mice per group. *p* values were calculated using an unpaired two‐tailed Student's *t*‐test for the indicated comparisons, with *p* < 0.05 considered statistically significant (ns, not significant).

To determine whether these synaptic changes were accompanied by alterations in neurotransmitter receptor expression, we examined the expression of several excitatory AMPA receptor subunits and inhibitory GABA(A) receptor subunits in the mouse PFC. qPCR analysis revealed decreased *GluR1*, *GluR2*, and *GluR4* expression in OE mice, and *GluR1* was restored in *Cntnap3*‐RE mice (**Figure** **7**A); while *Gabra3* and *Gabrb2* expression was increased in OE mice, *Gabrb2* was restored in *Cntnap3*‐RE mice (Figure 7B). Western blot confirmed decreased *GluR1* expression and increased *Gabrb2* in OE mice, which was restored in *Cntnap3*‐RE mice (Figure 7C,D). Finally, dendritic spine analysis showed no significant changes in spine density or morphology in either OE or *Cntnap3*‐RE neurons (Figure 7E), suggesting that *PAXIP1‐AS1* and *Cntnap3* primarily influences postsynaptic receptors rather than structural synapse formation.

### 2.6 PAXIP1‐AS1 Enhances CNTNAP3 Expression by Sequestering ZGPAT in an Allele‐Specific Manner

**Figure 8:**
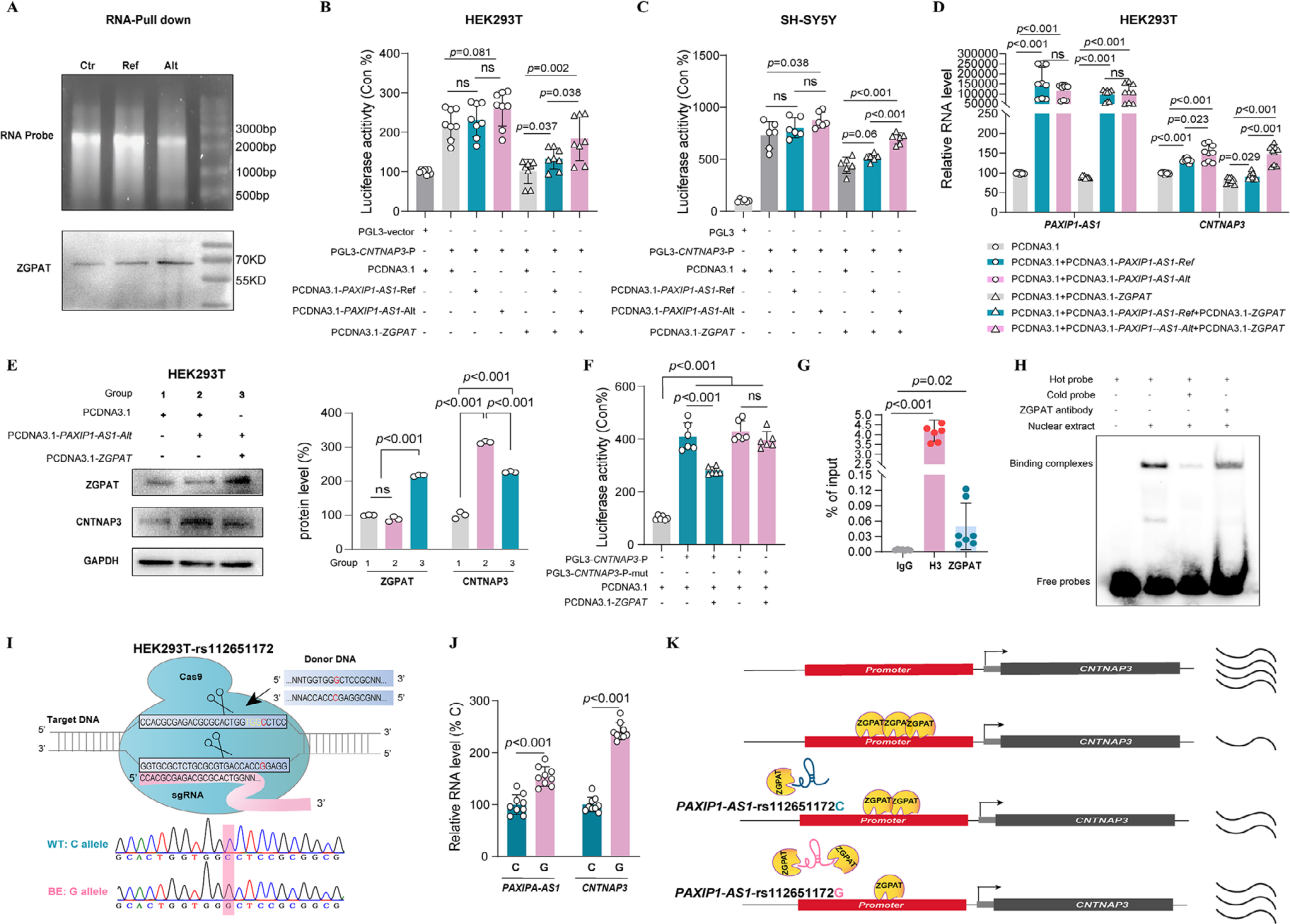
PAXIP1‐AS1 enhances CNTNAP3 expression by sequestering ZGPAT in an allele‐specific manner. A) RNA pull‐down assay showing ZGPAT binding to lncRNA‐PAXIP1‐AS1. Biotinylated control RNA, PAXIP1‐AS1‐Ref (Ref), or PAXIP1‐AS1‐Alt (Alt) (top) were incubated with HEK293T cell lysates and detected by ZGPAT immunoblotting (bottom). B,C) Allele‐dependent regulation of CNTNAP3 promoter activity in PLG3‐luciferase reporter in HEK293T (B) and SH‐SY5Y cells (C). D) qPCR analysis of endogenous CNTNAP3 mRNA expression in HEK293T cells following PAXIP1‐AS1 and ZGPAT OE. E) Western blot analysis confirming CNTNAP3 protein upregulation by PAXIP1‐AS1‐Alt OE and inhibition by ZGPAT OE in HEK293T cells. F) Luciferase reporter assays evaluating the regulatory effects of ZGPAT‐binding motif‐containing sequences from the CNTNAP3 promoter in HEK293T cells. G) ChIP‐qPCR analysis quantifying ZGPAT enrichment in the CNTNAP3 promoter regions in HEK293T cells. H) EMSA competition assay using probes containing ZGPAT‐binding motif sequences in HEK293T nuclear extracts, validated by anti‐ZGPAT gel shift assays. I,J) Schematic of HEK293T cell lines homozygous for either rs112651172C/C or rs112651172G/G generated via single‐base editing (I), and qPCR analysis of PAXIP1‐AS1 and CNTNAP3 expression in these cell lines (J). K) Proposed model: PAXIP1‐AS1 enhances CNTNAP3 expression by sequestering ZGPAT, preventing its binding to the CNTNAP3 promoter. This regulation is allele‐specific, with the Alt allele exhibiting stronger ZGPAT binding and greater *CNTNAP3* upregulation than the Ref allele. Data obtained from at least three independent experiments are presented as mean ± SD. *p* values were calculated using an unpaired two‐tailed Student's *t*‐test for the indicated comparisons, with *p* < 0.05 considered statistically significant (ns, not significant).

LncRNAs regulate gene expression by interacting with RNA‐binding proteins (RBPs). We used the catRAPID algorithm to determine whether *PAXIP1‐AS1* exerts allele‐specific regulatory effects through RBPs. Among the predicted RBPs, ZGPAT exhibited the most significant differential binding between the Alt and Ref alleles of *PAXIP1‐AS1* (Table S7, Supporting Information). RNA‐Pulldown assays confirmed that ZGPAT binds more strongly to the Alt allele than to the Ref allele (**Figure** **8**A). ZGPAT is known to repress gene expression by binding to GGAG[GA]A[GA]A‐like motifs in promoter or enhancer regions. Consistent with this, ZGPAT‐binding motifs were identified in the *CNTNAP3* upstream region, suggesting that *PAXIP1‐AS1* may enhance *CNTNAP3* expression by sequestering ZGPAT and preventing its binding to the *CNTNAP3* promoter. We hypothesize that allele‐specific differences in ZGPAT binding influence *CNTNAP3* regulation.

To test this, we conducted luciferase reporter assays in HEK293T and SH‐SY5Y cells co‐transfected with either *PAXIP1‐AS1‐*Alt or *PAXIP1‐AS1‐*Ref, a ZGPAT‐overexpression plasmid, and a pGL3 luciferase vector driven by the *CNTNAP3* promoter containing ZGPAT‐binding motifs. ZGPAT overexpression significantly reduced *CNTNAP3* promoter activity (Figure 8B,C), while *PAXIP1‐AS1* overexpression reversed this effect in an allele‐dependent manner. qPCR further confirmed that ZGPAT overexpression reduced *CNTNAP3* mRNA expression in a *PAXIP1‐AS1* allele dependent manner (Figure 8D). Western blot showed that *PAXIPA‐AS1* overexpression increased endogenous CNTNAP3 protein levels, whereas ZGPAT overexpression counteracted this effect (Figure 8E).

To confirm direct promoter binding, we deleted the ZGPAT‐binding region within the *CNTNAP3* promoter cloned into the pGL3 luciferase reporter construct. Deletion of this region significantly enhanced *CNTNAP3* promoter activity and abolished the repressive effect of ZGPAT overexpression, supporting a direct repressive role of ZGPAT (Figure 8F). Chromatin immunoprecipitation (ChIP‐qPCR) confirmed ZGPAT binding to the *CNTNAP3* promoter in HEK293T cells (Figure 8G), while electrophoretic mobility shift assays (EMSA) revealed ZGPAT‐binding complexes that were super‐shifted upon incubation with a ZGPAT antibody (Figure 8H). Finally, to directly assess the effect of rs112651172C>G on *PAXIP1‐AS1* expression, we generated HEK293T cell lines homozygous for either rs112651172C/C or rs112651172G/G using single‐base editing (Figure 8I). qPCR analysis revealed that the alternative G allele significantly upregulated both *PAXIP1‐AS1* and *CNTNAP3* expression (Figure 8J). Together, these findings suggest that *PAXIP1‐AS1* enhances *CNTNAP3* expression by sequestering the transcriptional repressor ZGPAT, thereby preventing its binding to the *CNTNAP3* promoter (Figure 8K). This mechanism is allele‐specific, with the Alt allele exhibiting stronger ZGPAT binding and greater *CNTNAP3* upregulation than the Ref allele.

### 2.7 Allele‐Specific Effects of rs112651172 on PAXIP1‐AS1 Expression via TF Binding

**Figure 9:**
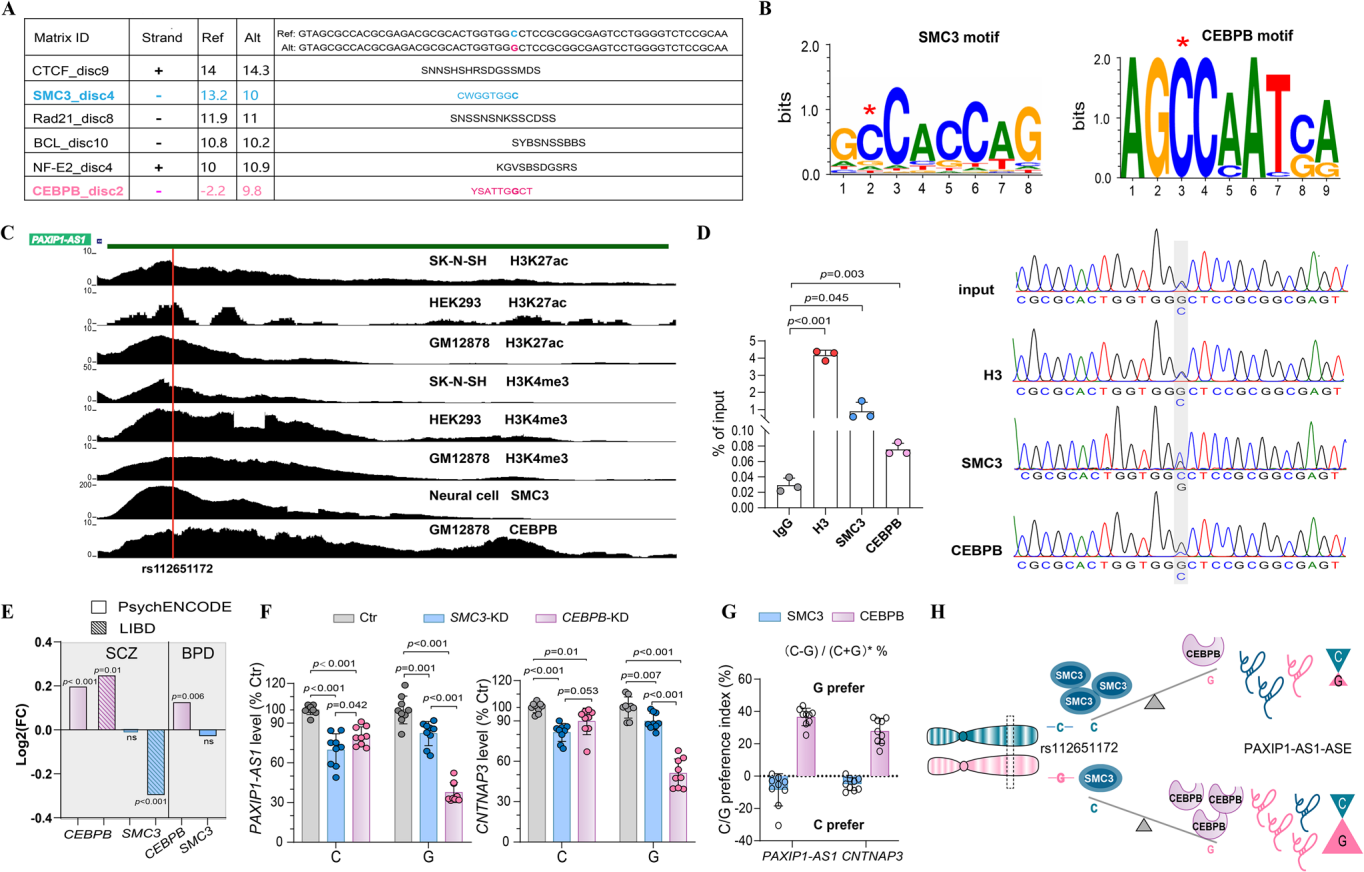
Regulation of PAXIP1‐AS1 expression by SMC3 and CEBPB at rs112651172 in an allele‐specific manner. A) HaploReg prediction of TFs with allele‐specific binding at rs112651172. B) Schematic of the SMC3 and CEBPB binding motifs. C) ChIP‐seq tracks from the ENCODE dataset show various histone marks and TF occupancy at the rs112651172 locus in SK‐N‐SH, HEK293T, GM12878, and Neural cells. D) ChIP analysis of SMC3 and CEBPB binding to rs112651172 in HEK293T cells with equal proportions of the two homozygous rs112651172 genotypes (C/C and G/G). Immunoprecipitated DNA was analyzed relative to input DNA (left), and sanger sequencing of PCR products from immunoprecipitated DNA containing the rs112651172 SNP was performed (right). E) SCZ‐ or BPD‐associated expression alterations of CEBPB and SMC3 as identified in the PsychENCODE and LIBD brain transcriptomic datasets. F,G) qPCR analysis of PAXIP1‐AS1 and CNTNAP3 expression in HEK293T cells homozygous for rs112651172C/C or rs112651172G/G (F), following KD of SMC3 or CEBPB. The C/G preference index shows the expression difference between rs112651172C/C and rs112651172G/G homozygous cells, normalized to the sum of expression and multiplied by 100 (G). H) Schematic illustrating the mechanism of PAXIP1‐AS1 regulation by SMC3 and CEBPB. Data obtained from at least three independent experiments are presented as mean ± SD. *p* values were calculated using an unpaired two‐tailed Student's *t*‐test, with *p* < 0.05 considered statistically significant (ns, not significant).

To investigate the regulatory effect of the rs112651172 (C/G) variant on *PAXIP1‐AS*1 expression, we used HaploReg to predict TFs with allele‐specific binding at this locus. The analysis identified SMC3 as preferentially binding to the Ref‐C allele, and CEBPB as favoring the Alt‐G allele (**Figure** **9**A,B). Supporting its regulatory potential, ENCODE histone modification data revealed that rs112651172 is located within actively transcribed regions in HEK293T and human neuroblastoma cells (Figure 9C top). Furthermore, ChIP‐seq data confirmed SMC3 enrichment at the rs112651172 locus in neural cells and CEBPB enrichment in GM12878 cells (Figure 9C bottom), providing additional evidence for a regulatory role of this variant. Consistent with these findings, ChIP assays in HEK293T cells transfected with *PAXIP1‐AS1* expression vectors harboring either C/C or G/G genotypes confirmed that SMC3 preferentially binds the C allele, while CEBPB binds the G allele (Figure 9D).

To assess the relevance of these TFs in psychiatric disorders, we analyzed their expression profiles using RNA‐seq data from PsychENCODE and LIBD brain transcriptomic datasets.^[^ ^21^, ^22^ ^]^ In the PsychENCODE dataset, *CEBPB* was significantly upregulated in both SCZ (log2FC = 0.1917, *P* = 1.05E‐8) and BPD (log2FC = 0.1257, *P* = 0.0061; Figure 9E). In contrast, *SMC3* showed slight but non‐significant downregulation (SCZ: log2FC = ‐0.0007, *P* = 0.9519; BPD: log2FC = −0.0286, *P* = 0.0842). These effects were further supported in the LIBD dataset, where *CEBPB* was also significant upregulated in SCZ patients (log2FC = 0.2481, *P* = 0.01; Figure 9E), and *SMC3* exhibited significant downregulation (log2FC = −0.2962, *P* = 5.15E‐8). The increased expression of CEBPB may contribute to the upregulation of *PAXIP1‐AS1* observed in SCZ and BPD brain tissues, as well as the Alt‐G allele‐dependent elevation seen in affected individuals of DC twin pars. These findings suggest that rs112651172 may modulate enhancer activity by altering TF binding, thereby regulating *PAXIP1‐AS1* expression in the context of psychiatric disorders. To further investigate this mechanism, we performed KD experiments in HEK293T cells with either the C/C or G/G genotype. Since both SMC3 and CEBPB are endogenously expressed at high levels in these cells, this model allowed us to mimic both normal and disease‐related expression states. KD of either CEBPB or SMC3 significantly reduced *PAXIP1‐AS1* and *CNTNAP3* expression, with allele‐specific effects. In C/C cells, SMC3 KD had a stronger effect, whereas in G/G cells, CEBPB KD led to greater downregulation (Figure 9F,G). These results support a model in which rs112651172 modulates enhancer activity via allele‐specific TF binding—SMC3 preferentially binding the C allele and CEBPB the G allele—thereby regulating *PAXIP1‐AS1* expression (Figure 9H). Additionally, dysregulation of these TFs in psychiatric disorders may further impact *PAXIP1‐AS1* and *CNTNAP3* expression, linking this regulatory variant to neuropsychiatric disease risk.

## 3 Discussions

This study presents a comprehensive analysis of ASE events in lncRNAs by integrating genomic and transcriptomic data from MZ twin pairs discordant for SCZ and BPD. We identify and functionally validate a key regulatory variant, rs112651172 (C/G), which mediates ASE of *PAXIP1‐AS1* through allele‐specific binding of SMC and CEBPB. This variant link noncoding genetic variation to psychiatric disease risk by modulating *PAXIP1‐AS1* and *CNTNAP3* expression, with downstream impacts on synaptic function and behavior. Together, these findings provide novel insights into how functional noncoding variants shape the genetic and epigenetic architecture of psychiatric diseases.

We propose a functional framework that connects noncoding genetic variants, ASE imbalance in lncRNAs, and their downstream molecular consequences relevant to psychiatric disorders. Although GWAS have identified numerous risk loci associated with SCZ and BPD,^[^ ^31^, ^32^, ^33^, ^34^, ^35^ ^]^ the majority of these loci reside in noncoding regions, and their regulatory functions remain poorly understood.^[^ ^36^, ^37^ ^]^ By leveraging MZ twin pairs discordant for psychiatric phenotypes, we identified ASE patterns of noncoding genetic variants that are unique to affected individuals compared to their unaffected co‐twins. This twin‐based approach allows for the detection of disease‐associated regulatory variation while controlling for genetic background. ASE‐associated SNPs were significantly enriched in open chromatin regions, suggesting them in the modulation of TF binding, chromatin accessibility, and epigenetic remodeling. Importantly, ASE switching between affected and unaffected twins highlights a dynamic interplay between genetic and epigenetic mechanisms. This functional asymmetry—observed in genetically identical individuals—provides a powerful readout of disease‐specific regulatory perturbations, extending beyond what can be inferred from genotype frequency alone. Among the 17 ASE‐associated lncRNA identified, five also showed differential expression in the PFC of SCZ or BPD patients in the PsychENCODE dataset, further strengthening the link between ASE and disease‐relevant transcriptional changes. Although previous studies have described ASE as a largely stochastic phenomenon in the human genome,^[^ ^38^, ^39^ ^]^ the presence of consistent ASE discordance linked to disease status in MZ twin pairs suggests a non‐random regulatory mechanism that may override baseline stochasticity. Together with prior evidence of allele‐specific DNA methylation and hydroxymethylation changes in psychiatric disorders,^[^ ^40^, ^41^ ^]^ our findings underscore the critical role of noncoding regulatory variants in shaping the transcriptional landscape and contributing to psychiatric disease risk.

We further characterized *PAXIP1‐AS1* as a key regulatory lncRNA whose expression is modulated by the rs112651172(C/G) variant through allele‐specific TF binding. Specifically, SMC3 preferentially binds the C allele, while CEBPB binds to the G allele, as supported by chromatin accessibility data and ChIP‐seq profiles from ENCODE. Functionally, these two TFs have distinct neurobiological roles. Our data suggest that increased CEBPB expression in psychiatric patients promotes G allele‐biased transcription of *PAXIP1‐AS1*, whereas decreased SMC3 expression reduces transcription from the C allele. Previous studies have implicated CEBPB in neuroinflammation, microglia activation, pyroptosis, and stress‐induced depressive behaviors.^[^ ^42^, ^43^, ^44^ ^]^ Additionally, stress‐induced glucocorticoids and pro‐inflammatory cytokines (e.g., IL‐1β, IL‐6, and TNF‐α) have been reported to directly upregulate CEBPB, while simultaneously inhibiting neuroprotective factors such as SMC3 or its regulatory factors.^[^ ^45^, ^46^, ^47^, ^48^ ^]^ Conversely, SMC3 is thought to exert neuroprotective effects by attenuating inflammatory responses and promoting neuronal stability.^[^ ^49^, ^50^ ^]^ Importantly, early‐life adversity is strongly associated with later psychotic and mood disorders, and is known to induce persistent pro‐inflammatory immune activation and microglial sensitization. This pro‐inflammatory state may facilitate sustained high expression of CEBPB.^[^ ^51^ ^]^ Together, these observations suggest that interaction between genetic susceptibility (e.g., allele‐specific binding at rs112651172) and environmental stressors (e.g., inflammation, stress‐induced cytokine signaling) may underlie the dysregulation of functionally antagonistic TFs such as CEBPB and SMC3 in SCZ and BPD, ultimately leading to allele‐biased lncRNA transcription. Further studies are warranted to explore these upstream regulatory mechanisms, particularly regarding how inflammatory environmental factors influence this gene‐environment interaction.

We then demonstrate that *PAXIP1‐AS1* exerts allele‐specific effects on behavior, cognition, and synaptic function. By mimicking the overexpression of the disease‐associated G allele specifically in the PFC of wild‐type mice, we found that *PAXIP1‐AS1*‐G overexpression induces a spectrum of behavioral abnormalities, including anxiety‐ and depression‐like behaviors, working memory deficits, sensorimotor gating impairments, and reduced social preference. The absence of significant effects in the EPM or Barnes Maze tests indicates that these behavioral disruptions are domain‐specific rather than reflective of generalized neurological or cognitive dysfunction. This specificity aligns with the molecular and synaptic alterations observed predominantly within the PFC circuitry. The PFC is critically involved in executive functions, working memory, social cognition, and sensorimotor gating ‒ domains directly reflected in the deficits identified through our OFT, Y‐maze, PPI, and social interaction assays. Conversely, anxiety‐like behavior in the EPM and spatial navigation assessed by the Barnes Maze are more dependent on amygdala‐ and hippocampal‐mediated pathways, respectively,^[^ ^52^, ^53^, ^54^ ^]^ and thus may be less directly impacted by PFC‐specific dysregulation of *PAXIP1‐AS1*, or potentially compensated by other intact circuits. This domain‐specific phenotype parallels the clinical heterogeneity observed in SCZ and BPD, where patients commonly exhibit pronounced deficits in certain cognitive and social domains, while other functions remain relatively spared.

Importantly, behavioral abnormalities were notably more pronounced in female mice, consistent with clinical observations of female‐specific upregulation of *PAXIP1‐AS1* expression in the PFC of patients with MDD, and with known sex‐biased vulnerability and symptom profiles in mood and psychotic disorders. This strong suggests the presence of sex‐dependent regulatory mechanisms influencing lncRNA function and psychiatric vulnerability. For instance, estrogen signaling plays a key role, as estrogen receptors (ERα/β) can directly bind to estrogen response elements (EREs) in lncRNA promoters^[^ ^55^, ^56^ ^]^ or interact with TFs such as CEBPB^[^ ^57^, ^58^ ^]^ to influence chromatin accessibility. Given the inflammatory roles of CEBPB which upregulated in SCZ and BPD and its known sensitivity to hormonal fluctuations, estrogen‐CEBPB interactions may further amplify G‐allele‐driven *PAXIP1‐AS1* expression specifically in females. Additionally, escape from X‐chromosome inactivation (XCI) could indirectly affect autosomal lncRNAs expression through trans‐acting X‐linked modifiers, including regulators of XIST regulators.^[^ ^59^ ^]^ These genetic and hormonal mechanisms, further compounded by environmental stressors that differentially engage neuroimmune pathways in females, may collectively heighten susceptibility to psychiatric disorders. Future studies are essential to clarify precisely how hormonal influences and sex‐chromosome interactions shape ASE patterns of *PAXIP1‐AS1* and related psychiatric risk loci.

At the molecular level, transcriptomic profiling revealed widespread gene expression changes in the PFC, with G allele overexpression leading to downregulation of genes associated with mitochondrial energy metabolism and protein complex assembly, and upregulation of pathways involved in neuronal development and ion transport. These transcriptional shifts are indicative of synaptic stress and functional remodeling, consistent with the observed behavioral phenotypes. Electrophysiological recordings further established that *PAXIP1‐AS1*‐G overexpression compromises neuronal excitability and disrupts excitatory/inhibitory (E/I) balance. Reductions in action potential firing, eEPSCs, and mEPSC amplitudes—alongside increased sEPSC frequencies and elevated inhibitory currents—point to a net decrease in excitatory signaling and possible compensatory inhibitory responses. These synaptic changes occur in the absence of altered dendritic spine density, indicating that functional rather than structural synaptic impairments underlie the phenotype.

Mechanistically, we identified *CNTNAP3* as a downstream effector of *PAXIP1‐AS1*. *CNTNAP3* is a known synaptic adhesion molecule with roles in both excitatory and inhibitory synapse development. We show that *CNTNAP3* expression is upregulated in response to *PAXIP1‐AS1*‐G overexpression across multiple systems (mouse PFC, HEK293T, and SK‐N‐SH cells), and its expression correlates with that of *PAXIP1‐AS1* in human brain data. Functional rescue experiments confirmed that *Cntnap3* knockdown in *PAXIP1‐AS1*‐overexpressing mice significantly reversed behavioral abnormalities and restored synaptic function, strongly supporting *CNTNAP3* as a key mediator of *PAXIP1‐AS1*‐induced pathology. Interestingly, *PAXIP1‐AS1* overexpression also led to the upregulation of inhibitory GABA(A) receptor subunits and the downregulation of excitatory AMPA receptor subunits, further implicating excitation/inhibitory (E/I) imbalance as a core feature of its downstream effect. These changes were partially reversed upon *Cntnap3* knockdown, reinforcing the model that *PAXIP1‐AS1*–*CNTNAP3* signaling axis regulates synaptic function and behavioral outputs through modulation of inhibitory circuitry.

Several limitations of this study should be acknowledged. First, ASE analysis was conducted in peripheral blood, which may not fully reflect the brain‐specific regulatory landscape, particularly for neurodevelopmental and psychiatric traits. Second, the limited number of discordant MZ twin pairs constrains the statistical power to detect low‐frequency or subtle ASE events. Third, while the PFC was selected based on its established involvement in psychiatric disorders, the role of *PAXIP1‐AS1* in other brain regions—such as the hippocampus, amygdala, or striatum—remains to be explored. Additionally, we observed that behavioral abnormalities were more pronounced in female mice, raising the possibility that sex‐specific hormonal or epigenetic factors may modulate the impact of ASE‐associated variants and should be investigated in future studies.

In conclusion, this study identifies rs112651172 as a functional noncoding variant that regulates *PAXIP1‐AS1* expression via allele‐specific TF binding, leading to downstream modulation of *CNTNAP3* and associated synaptic and behavioral abnormalities relevant to SCZ and BPD. By integrating genomic analysis of discordant MZ twins, molecular validation, and in vivo functional modeling, we establish a mechanistic link between noncoding genetic variation and psychiatric disease phenotypes. These findings underscore the pathogenic relevance of allele‐specific lncRNA regulation and highlight a novel regulatory axis (*PAXIP1‐AS1*–*CNTNAP3*) with potential as a target for precision psychiatry interventions.

## 4 Experimental Section

### Twin Subjects

Nine pairs of DC MZ twins (18 individuals), including four SCZ DC twins (SDC) and five BPD DC twins (BDC), were recruited in this study (Table S1, Supporting Information). The zygosity was determined by the Qiagen Investigator Argus X‐12 QS Kit (USA). All patients fulfilled the diagnostic criteria of SCZ or BPD according to the Diagnostic and Statistical Manual of Mental Disorder, 5th Edition (American Psychiatric Association). All participants in this study provided informed consent prior to the study following presentation of the nature of the procedures. The study was approved by the Medical Ethics Committee of Zhujiang Hospital of Southern Medical University (#2022‐KY‐086), Guangdong Provincial People's Hospital (#KY2024‐915‐02), and the third People's Hospital of Zhongshan (SSYLL20210301) and conducted in accordance with the Declaration of Helsinki. Genomic DNA and RNA were isolated from peripheral blood using the standard phenol‐chloroform method for whole‐genome sequencing (WGS) and whole‐transcriptome sequencing.

### WGS and Genotyping

To obtain genome‐wide SNP genotype, WGS was performed on DNA from unaffected con‐twins of 9 MZ twin pairs. Sequencing was conducted by Novogene (Tianjin, China) using the Illumina Nova‐seq platform, generating ≈700 million 150‐bp paired‐end reads per sample. Raw sequencing data quality was assessed using FASTQC (https://www.bioinformatics.babraham.ac.uk/projects/fastqc/), and reads were aligned to the human reference genome (hg19) using BWA.^[^ ^60^ ^]^ Duplicate reads were removed using Picard, and base quality score recalibration was performed using BaseRecalibrator and ApplyBQSR in GATK4,^[^ ^61^ ^]^ with reference to public variant datasets (1000 Genomes phase 1, Mills & 1000G gold standard indels, and dbSNP build 146). Variant calling was performed using HaplotypeCaller in GATK4. Variant quality score recalibration and filtering were applied using VariantRecalibrator and ApplyVQSR, respectively, to produce high‐confidence SNP genotypes.

SNP annotation was carried out using ANNOVAR,^[^ ^62^ ^]^ focusing on SNPs located within exonic regions of lncRNA transcripts. Genotype files were integrated with annotation data to construct SNP‐lncRNA transcript pairs. Among the 11773 lncRNA transcripts in the genome, 7698 contained at least one SNP that was heterozygous in at least one of the 9 PDC MZ twin pairs, yielding a total of 20817 unique SNPs. From these, 25840 SNP‐lncRNA transcript pairs were derived for downstream analysis.

### Haplotype Quantification at the Transcriptomic Level

Using the SNP genotype files, haplotypes for each MZ twin were initially constructed with SHAPEIT, incorporating reference haplotype data from the Han Chinese population in the 1000 Genomes Project. Individualized haplotype‐resolved reference genomes were then generated using g2gtools and vcf2doploid, allowing the creation of two personalized genome sequences per individual, each representing one haplotype. For RNA‐seq data, quality control was performed using FASTQC on paired‐end reads obtained from 9 pairs of PDC twins (18 individuals). Clean reads were aligned separately to the two haplotype‐specific reference genomes for each individual using Bowtie2.^[^ ^63^ ^]^ Haplotype‐level transcript quantification was then carried out using the EMASE (Expectation‐Maximization algorithm for Allele Specific Expression) algorithm (https://github.com/churchill‐lab/emase). EMASE‐generated expected read counts were subsequently used for ASE analysis.

### Psychiatry‐Associated ASE Analysis

For each PDC MZ twin pair, EMASE‐derived expected read counts for the two haplotypes were integrated at the transcript level, leveraging the fact that affected and unaffected co‐twins share an identical personalized reference genome. Fisher's exact test was performed to identify differential haplotype expression between affected and unaffected individuals. Psychiatry‐associated ASE genes were initially screened using a false discovery rate (FDR) threshold of <0.1, and these were further integrated with SNP‐lncRNA transcript pairing data.

ASE results were next combined across the 9 MZ twin pairs. Due to individual haplotype differences, exon‐overlapping SNPs within lncRNAs were used to align haplotype‐based expected counts across all 18 individuals. Because multiple SNPs can exist within a single transcript, haplotype quantification could vary across SNPs for the same lncRNA. This analysis yielded 2016 SNPs corresponding to 531 transcripts, forming 2151 SNP‐transcript pairs. From these, 754 pairs SNP–comprising 726 SNPs and 219 transcripts–exhibited consistent disease‐related ASE changes in at least two twin pairs.

To further prioritize SNP–lncRNA transcript pairs with consistent psychiatry‐associated ASE transitions, Bayesian inference was performed using the R package INLA (www.r‐inla.org). The Bayesian factors (BF) compared the likelihood of two models:

1. Model 1 (M1): logit(*p*_is_) = β_0_ + β_s_ + γ_i_ [Includes a phenotype effect (βs) on ASE]
2. Model 0 (M0): logit(*p*_is_) = β_0_ + γ_i_ [No phenotype effect (only intercept and sample effect)]

Here, *p_is_* denotes the proportion of reads supporting the alternative allele for individual *i* with disease status *s*, *β_s_* represents the phenotype effect; and *γ_i_* is a random effect accounting for inter‐individual variability.

BF quantifies the evidence in favor of phenotype‐specific ASE (i.e., M1 over M0). A higher BF indicates stronger evidence that ASE is associated with disease status.

1. BF >100: Strong support for phenotype‐specific ASE (robust differential allelic imbalance)
2. BF <0.01: Strong support for ASE without phenotype specificity
3. 1/3 <BF <3: Inconclusive—insufficient evidence to distinguish between models.

A stringent threshold of BF >100 was applied, identifying 283 high‐confidence SNP‐transcript pairs, corresponding to 277 SNPs and 104 transcripts, showing robust and consistent ASE differences linked to psychiatric status.

### Enrichment Analysis of ASE SNPs

To assess whether the 277 ASE‐SNPs were enriched in regulatory functions, enrichment analyses were performed using 18801 control SNPs that did not show both ASE and heterozygosity in any twin pair. Two complementary datasets were employed: 1) RegulomeDB^[^ ^18^, ^64^ ^]^ annotation and 2) motifbreakR^[^ ^19^, ^65^ ^]^ prediction of transcript factor (TF) binding disruption. Statistical significance was assessed using Fisher's exact test.

RegulomeDB‐based annotation: RegulomeDB classifies SNPs into regulatory categories ranging from type 1 to 7, with lower type numbers indicating stronger evidence for regulatory function (Table S3, Supporting Information). Categories 1–4 are supported by multiple lines of functional evidence (e.g., eQTL, TF binding, DNase peaks) and are considered likely regulatory variants. All ASE SNPs were annotated using the RegulomeDB database, and the number of SNPs falling into each regulatory type was quantified. enrichment of ASE SNPs among types 1–4 was then tested relative to the control SNPs.

TF Binding Disruption (motifbreakR): To predict the potential impact of SNPs on TF binding, motifbreakR was applied. Given that a single SNP can affect binding of multiple TFs, SNPs were categorized into two groups: those predicted to disrupt at least one TF binding site and those with no predicted TF binding disruption. It is then tested whether ASE SNPs were significantly more likely to disrupt TF binding compared to control SNPs. Gene Ontology enrichment analyses were performed of these gene sets using the web‐based ToppGene Suite.^[^ ^66^ ^]^

Together, these analyses aimed to determine whether ASE‐associated SNPs are preferentially located in regions of functional regulatory activity, supporting their potential roles in gene regulation relevant to psychiatric phenotypes.

### Cell Culture

The human embryonic kidney cell line HEK293T was cultured in Dulbecco's Modified Eagle Medium (DMEM; Life Technologies, USA) supplemented with 10% fetal bovine serum (FBS). The human neuroblastoma cell line SK‐N‐SH was maintained in DMEM supplemented with 1× MEM Non‐Essential Amino Acids (NEAA), 1× GlutaMAX Supplement, and 10% FBS. The SH‐SY5Y neuroblastoma cell line was grown in Dulbecco's Modified Eagle Medium/Nutrient Mixture F‐12 (DMEM/F‐12; Life Technologies, USA) supplemented with 10% FBS. All cell lines were cultured at 37 °C in a humidified incubator with 5% CO_2_.

### Recombinant Plasmid Construction

To generate expression constructs, the full‐length cDNA of *PAXIP1‐AS1* (ENSG00000273344.2) harboring either the reference C allele or the alternative G allele at rs112651172 (C/G) was cloned into the pcDNA3.1(+) expression vector under the control of the CMV promoter, resulting in pcDNA3.1‐*PAXIP1‐AS1‐C* and pcDNA3.1‐*PAXIP1‐AS1‐G*, respectively. Similarly, cDNA sequences corresponding to *CEBPB* (ENST00000303004.5), *SMC3* (ENST00000361804.5), and *ZGPAT* (ENST00000328969.5) were each cloned into the pcDNA3.1(+) expression vector under the control of the CMV promoter to generate the expression plasmids pcDNA3.1‐*CEBPB*, pcDNA3.1‐*SMC3*, and pcDNA3.1‐*ZGPAT*.

For reporter assays, the *PAXIP1‐AS1* promoter region was cloned into the pGL4.18 dual‐luciferase reporter vector (Promega). A 2460 bp genomic fragment spanning 1780 bp upstream to 680 bp downstream of the *PAXIP1‐AS1* transcription start site (chr19:42786783–42788862; GRCh37/hg19) was inserted via NheI and BamHI restriction sites, yielding pGL4.18‐*PAXIP1‐AS1*‐p. The *CNTNAP3* promoter region was cloned into the pGL3‐Basic vector (Promega) using KpnI and XhoI restriction sites. A 2080 bp reverse strand of genomic fragment spanning from 1780 bp upstream to 300 bp downstream of the *CNTNAP3* transcription start site (chr9:39284880–39289200; GRCh37/hg19) was used to generate pGL3‐*CNTNAP3*‐p.

### Recombinant Adeno‐Associated Virus (rAAV) Packaging

To generate rAAV constructs expressing lncRNA *PAXIP1‐AS1* with either allele of rs112651172, the full‐length cDNA of *PAXIP1‐AS1* (ENSG00000273344.2) containing the reference C allele or the alternative G allele was cloned into the rAAV‐hSyn‐EGFP‐WPRE‐bGH polyA vector under the human synapsin (hSyn) promoter, resulting in rAAV‐*PAXIP1‐AS1*‐C and rAAV‐*PAXIP1‐AS1*‐G, respectively. An empty rAAV vector (AAV‐EV) with no insert was used as a negative control. All constructs included an EGFP reporter gene driven by the hSyn promoter. rAAV amplification and purification were performed by Shumi Biotechnology (Wuhan, China) according to the manufacturer's protocol.

To knock down mouse *Cntnap3*, an AAV‐shRNA construct (AAV‐sh‐*Cntnap3*) was generated by inserting a short hairpin RNA (shRNA) targeting the mouse *Cntnap3* mRNA into the pAAV‐U6‐shRNA‐CMV‐mScarlet‐WPRE expression vector. A non‐targeting shRNA (AAV‐sh‐NC) was used as a negative control. In both constructs, the shRNA was driven by the U6 promoter, and a mScarlet fluorescent reporter was driven by the CMV promoter. AAV amplification and purification were carried out by Obio Technology (Shanghai, China) following standard protocols. The shRNA sequence targeting mouse *Cntnap3* mRNA, CAGACAGTGTGGTACAATA, was reported in a previous study.^[^ ^26^ ^]^

### Mouse Behavior Tests

C57BL/6 mice were obtained from the Southern Medical University Experimental Animal Center (Guangdong, China). Mice were housed in groups under a standard 12‐h light/dark cycle with ad libitum access to food and water. All behavioral experiments were conducted during the light phase. All procedures were approved by the Ethics Committee of Guangdong General Hospital (KY2024‐915‐02).

### Stereotaxic Microinjection

Mice were anesthetized and positioned in a digital stereotaxic frame with ear bars and a nose clamp. Following a midline scalp incision, a cranial burr hole was drilled at stereotaxic coordinates targeting the prefrontal cortex (PFC; Paxinos coordinates: AP = +1.80 mm, ML = ±0.30 mm, DV = −1.90 mm from dura). Bilateral microinjections of AAV (1.0 µL per side) were delivered using a 33‐gauge Hamilton microsyringe at a rate of 0.1 µL min^−1^. The needle was left in place for 3 min post‐infusion to allow for viral diffusion before slow retraction. Mice were allowed to recover on heating pads and received postoperative analgesia. Behavioral testing was conducted 4 weeks after AAV injections.

### Spontaneous Locomotor Activity

Mice were placed in the center of a rectangular chamber (40 × 40 × 30 cm) made of gray polyvinyl chloride and allowed to freely explore for 5 min. Locomotor behavior was recorded with an automated video tracking system (EthoVision 11.0, Noldus) and analyzed for total distance traveled, time spent in the central zone, and jump counts. The apparatus was cleaned with 75% ethanol and air‐dried between trials to remove olfactory cues.

### Elevated Plus Maze (EPM)

Anxiety‐related behaviors were assessed using an elevated plus maze consisting of two open arms (30 × 5 cm), two closed arms (30 × 5 × 15 cm), and a central platform (5 × 5 cm) elevated 50 cm above the floor. Each mouse was placed in the center of the maze facing an open arm and allowed to explore for 5 min. Time spent in each arm was recorded using EthoVision 11.0. The maze was cleaned with 75% ethanol and allowed to air‐dry between animals.

### Forced Swim Test (FST)

Depressive‐like behavior was measured using the forced swim test. Mice were placed individually in a transparent glass cylinder (height: 45 cm, diameter: 19 cm) filled with 23 cm of water (23 ± 1 °C) for 6 min. Immobility time was recorded during the last 4 min. Tests were performed in a temperature‐controlled environment (25 ± 1 °C).

### Three‐Chamber Social Test

Sociability and social novelty preference were assessed in a three‐chamber apparatus. The test consisted of three phases: 10‐min habituation, 10‐min social approach, and 10‐min social novelty preference. In the social approach phase, an unfamiliar conspecific (Stranger 1) was placed in a wire cage in one side chamber, while the opposite chamber contained an empty cage. For social novelty testing, a novel mouse (Stranger 2) was introduced into the previously empty cage. Time spent in each chamber was recorded automatically.

### Y‐Maze Test

Spontaneous alternation behavior was assessed in a Y‐maze with three arms (40 × 15 × 15 cm). Mice were placed in the center and allowed to explore for 5 min. The sequence and total number of arm entries were recorded, and the percentage of correct spontaneous alternation was calculated.

### Prepulse Inhibition (PPI)

Sensorimotor gating was assessed using an SR‐LAB startle system (San Diego Instruments, CA, USA). Mice were placed in Plexiglas cylinders on a motion‐sensitive platform. After a 5‐min habituation with 70 dB background noise, mice were exposed to six startle trials (120 dB white noise, 40 ms) and six prepulse trials (20 ms at 74, 78, or 82 dB followed by a 100 ms delay and 120 dB startle pulse). Trials were presented pseudorandomly. PPI was calculated as:

PPI (%) = ((Vmax_startle − Vmax_PP) / Vmax_startle) × 100%.

### Barnes Maze

Spatial learning and memory were tested using the Barnes maze (100 cm diameter, Ugo Basile, Italy) surrounded by 20 holes divided equally into four quadrants by adjacent locations, with a hidden escape box under a random hole in one of the quadrants. Visual cues were placed on the surrounding walls, and bright lighting (≈1000 lx at the center) provided aversive motivation. Mice were trained over five days. On Day 1, mice were guided to the escape hole and allowed to remain for 120 s. On Days 2–6, mice explored freely for 180 s; if they did not find the escape hole, they were gently guided to it. For each mouse, the location of the escape hole was kept in the same quadrant. A probe trial was performed 24 h after the last training to assess memory retention. Latency to find the escape hole was recorded using video tracking and manual scoring.

### Slice Preparation

Eight‐week‐old mice were anesthetized with pentobarbital and decapitated. Brains were rapidly removed and submerged in ice‐cold oxygenated dissection buffer containing: sucrose (195 mm), KCl (2 mm), CaCl_2_ (0.2 mm), MgSO_4_ (12 mm), NaH_2_PO_4_ (1.3 mM), NaHCO_3_ (26 mm), and glucose (10 mm). Coronal slices (300 µm) encompassing the prefrontal cortex (PFC) were prepared using a vibratome (VT1200, Leica, USA). Slices were transferred to a holding chamber containing standard continuously oxygenated artificial cerebrospinal fluid (ACSF) composed of: NaCl (125 mm), KCl (2.5 mm), NaHCO_3_ (25 mm), NaH_2_PO_4_ (1.25 mm), glucose (30 mm), CaCl_2_ (2.0 mm), and MgCl_2_ (1.0 mm), with pH ≈7.3 and osmolarity ≈295 mOsm. Slices were incubated at 34 °C for 30 min to allow recovery and then maintained at room temperature (20–25 °C) for an additional 30 min before recordings. Patch pipettes (3.5–5 MΩ) were pulled from borosilicate glass capillaries using a micropipette puller (P‐100, Narishige, Japan).

### Whole‐Cell Patch‐Clamp Recordings

Coronal brains were transferred to a recording chamber perfused with oxygenated ACSF at 2–3 mL min^−1^ and maintained at 30–32 °C. Whole‐cell patch‐clamp recordings were conducted using an inverted microscope (Nikon, Japan) equipped with epifluorescence and infrared differential interference contrast (IR‐DIC) optics. A 4× air and 40× water immersion objective was used for locating and targeting cells. Signals were recorded with an EPC 10 USB patch‐clamp amplifier (HEKA Elektronik, Germany).

Neurons selected for whole‐cell recordings exhibited an initial leak current <30 pA and an access resistance <25 MΩ. Series resistance was monitored in each sweep using the peak amplitude of capacitance transients evoked by a −5 mV, 40 ms hyperpolarizing step pulse; only cells showing <20% change throughout the recording were included.

To assess the excitability of PFC pyramidal neurons, recording pipettes were filled with an internal solution containing: K‐gluconate (135 mm), MgCl_2_ (1 mm), HEPES (10 mm), EGTA (0.2 mm), Na_2_‐phosphocreatine (4 mm), Mg‐ATP (3 mm), and Na‐GTP (0.3 mm), with pH ≈7.2 and osmolarity ≈290 mOsm. After obtaining the whole‐cell configuration, resting membrane potential (RMP), membrane resistance (Rm), and capacitance (Cm) were measured. RMP was adjusted to −60 mV before injecting step currents from 0 to 400 pA in 50 pA increments at 10 s intervals.

For recordings of evoked or spontaneous excitatory and inhibitory postsynaptic currents (eEPSCs/eIPSCs or sEPSCs/sIPSCs), recording pipettes were filled with a cesium‐based internal solution containing: CsMeSO_3_ (130 mm), NaCl (4 mm), HEPES (10 mm), QX314 (5 mm), Mg‐ATP (4 mm), Na‐GTP (0.3 mm), and EGTA (0.5 mm), with pH ≈7.3 and osmolarity ≈290 mOs. To isolate excitatory synaptic currents (eEPSCs or sEPSCs), neurons were voltage‐clamped at −60 mV, a verified chloride reversal potential. Inhibitory synaptic currents (eIPSCs or sIPSCs) were recorded at a holding potential of +10 mV, corresponding to the reversal potential for AMPA and NMDA receptor‐mediated currents. For evoked recordings (eEPSCs and eIPSCs), synaptic responses were elicited by a 0.1 ms electrical pulse delivered every 15 s through a concentric bipolar stimulation electrode positioned near the recorded cell. To assess miniature postsynaptic currents (mEPSCs and mIPSCs), tetrodotoxin (TTX, 1 µm) was added to the ACSF to block action potential‐dependent neurotransmission. Holding potentials for mEPSC and mIPSC recordings were set to −60 and +10 mV, respectively, consistent with the conditions used for sEPSC and sIPSC recordings.

Data acquisition was performed using PatchMaster software (HEKA Elektronik, Germany), and subsequent data analysis and processing were conducted using AxonGraphX (AxonGraph Scientific, Australia).

### Confocal Imaging and Dendrite Spine Analysis

Fluorescent dendrite images of prefrontal cortical pyramidal neurons were captured using a Laser scanning confocal microscope (Nikon A1R HD25, Japan). Images were acquired in sequential scanning mode under a 63× oil immersion objective lens with 4× zoom at an optical slice thickness of 0.2 µm intervals along the *z*‐axis, with an image resolution of 1024 × 1024 pixels.

Neurons selected for dendritic spine analysis met the following criteria^[^ ^67^ ^]^: (I) minimal overlap with neighboring labeled cells; (II) visibility of at least three primary dendrites, and (III) presence of distal dendritic segments. Spine density was quantified from 1–4 dendritic segments (∼30 µm in length) on third‐order dendrites. For each experimental group, 3–6 neurons per mouse were analyzed. Dendritic protrusions were categorized into three morphological subtypes with slight modifications from previous criteria^[^ ^68^ ^]^: class 1, stubby spines, length <0.5 µm with no discernible head; class 2, mushroom‐shaped spines, head diameter >0.5 µm or >2× neck diameter; class 3, thin spines, length 0.5–3.0 µm with head diameter <0.5 µm. All measurements were made with Microscopy Image Analysis Software (Imaris 10.2, Switzerland).

### Target DNA Editing

CRISPR‐based genome editing was employed to introduce the rs112651172 C‐to‐G substitution within the exon of the *PAXIP1‐AS1* gene. Guide RNA (gRNA) sequences and donor repair templates were designed using the Zhang Lab CRISPR design tool (https://zlab.bio/guide‐design‐resources). The gRNA was cloned into a BsmBI‐digested pGL3‐U6‐sgRNA‐PURO entry vector under the control of the U6 promoter. The Cas9 endonuclease was expressed from a pcDNA3.3‐hCas9‐GFP plasmid driven by the CMV promoter.

HEK293T cells were co‐transfected with the pGL3‐U6‐sgRNA‐PURO and pcDNA3.3‐hCas9‐GFP plasmids using a standard lipid‐mediated transfection protocol. 5 h post‐transfection, cells were transfected with the single‐stranded donor oligonucleotide (ssODN) repair template containing the desired C‐to‐G substitution. Forty‐eight hours after transfection, GFP‐positive cells were isolated by fluorescence‐activated cell sorting (FACS) and seeded into 96‐well plates to establish monoclonal cell lines. Genomic DNA was extracted from expanded clones and subjected to Sanger sequencing to confirm successful editing of rs112651172.

### Chromatin Immunoprecipitation (ChIP)

ChIP assays were performed using cell lysates derived from two experimental systems. First, HEK293T cells overexpressing ZGPAT were subjected to immunoprecipitation using an anti‐ZGPAT antibody (SC‐515524, Santa Cruz Biotechnology). Second, HEK293T cells with homozygous rs112651172 C/C and G/G genotypes were mixed at a 1:1 ratio and processed for ChIP using anti‐CEBPB (23431‐1‐AP, Proteintech) and anti‐SMC3 (2845882, Invitrogen) antibodies.

Chromatin isolation, immunoprecipitation, and DNA purification were performed following the manufacturer's protocol using the EZ‐ChIP Kit (Merck, Germany). Immunoprecipitated DNA was subsequently analyzed by qPCR to assess transcription factor binding at target genomic regions.

### Western Blot Analysis

Prefrontal cortex tissues and cultured cells were homogenized in RIPA lysis buffer (Beyotime, P0013B) supplemented with proteinase inhibitors (Solarbio, Beijing, China). Protein samples (20–40 µg) were separated by 10–12% SDS‐PAGE and transferred onto polyvinylidene fluoride (PVDF) membranes (Millipore, IPVH00010). The membranes were blocked with 5% nonfat milk for 1 h at room temperature and then incubated overnight at 4 °C with primary antibodies: CNTNAP3 (Ab252413, Abcam), ZGPAT (SC‐515524, Santa Cruz Biotechnology), GluR1 (67642‐1‐Ig, Proteintech), GABRB2 (H681506024, HUABio), and GAPDH (60004‐1‐Ig, Proteintech).

After washing three times with TBS‐T (Tris‐buffered saline with 0.1% Tween 20), the membranes were incubated with horseradish peroxidase‐conjugated secondary antibodies at room temperature for 1 h. Protein signals were detected using a Tanon 5200 digital image scanner and quantified using ImageJ software.

### Quantitative Real‐Time PCR

Total RNA was extracted from cell lines or mouse prefrontal cortex (PFC) tissues using TRIzol reagent (Invitrogen). cDNA was synthesized from 1 µg of RNA using the PrimeScript RT Reagent Kit with gDNA Eraser (Takara, China), following the manufacturer's instructions.

Quantitative PCR was performed using SYBR Green dye‐containing SuperArray PCR Master Mix (Takara) on a Roche LightCycler 96 system. GAPDH was used as a reference gene to normalize the expression of target genes. The relative gene expression levels were calculated using the comparative ΔΔCt method, and data were subjected to statistical analyses. Total RNA extracted from cell lines or mouse PFC brains using TRIzol reagent (Invitrogen) was reversely transcripted to cDNA using a PrimeScript RT Reagent Kit with gDNA Eraser (Takara, China). A comparative qPCR assay with SYBR green dye‐containing SuperArray PCR master mix (Takara) was performed on an Roche LightCycler 96 with GAPDH as a reference gene for the quantification of target mRNA levels and the estimated values expressed as 2^−ΔΔCt^ were subjected to statistical analyses. The Primers and probes employed in this study are detailed in Table S8 (Supporting Information).

### Electrophoretic Mobility Shift Assay (EMSA)

Nuclear extracts were prepared from HEK293T cells and subjected to EMSA to assess the binding affinity of ZGPAT protein to the ZGPAT binding motif‐containing sequences in the *CNTNAP3* promoter. The assay was conducted using a Chemiluminescent Nucleic Acid Detection Module Kit (Thermo Fisher, USA). For supershift assays, a ZGPAT antibody (SC‐515524, Santa Cruz Biotechnology) was included to confirm the specificity of the protein‐DNA interaction. Biotin‐labeled DNA (Life Technologies) complexes were detected using a chemiluminescence imaging system (Tanon 5200).

### RNA‐Pulldown Assay

The full‐length lncRNAs were synthesized in vitro using a Ribo RNAmax‐T7 Transcription Kit (RiboBio, China). LncRNA‐*PAXIP1‐AS1* containing the reference C allele (Ref), lncRNA‐*PAXIP1‐AS1* containing the alternative G allele (Alt), and the antisense of lncRNA‐*PAXIP1‐AS1* containing the reference C allele (Ctr) were desthiobiotinylated using Pierce RNA 3′ End desthiobiotinylation Kit (20163, Thermo Scientific, USA). The RNA pull‐down assay was performed using a ‌Pierce Magnetic RNA‐Protein Pull‐Down Kit (20164Y, Thermo Scientific, USA). Briefly, desthiobiotinylated lncRNA was conjugated to streptavidin‐labeled magnetic beads. The HEK293T cell lysate was then added, and the mixture was incubated for 2 h at 25 °C. The magnetic beads were collected using a magnetic holder and washed. The collected proteins were analyzed by immunoblotting using a ZGPAT antibody to detect binding.

### Statistical Analysis

Statistical analyses were performed using GraphPad Prism software (version 8.0, GraphPad Software). Two‐tailed Student's *t*‐test, and ANOVA were performed, as appropriate. All experiments that undergo error analysis were carried out in at least three independent replicates. Data were presented with the mean ± standard deviation (SD)/standard error (SE).

### Compliance with Ethics

The study was approved by the Medical Ethics Committees of Zhujiang Hospital (#2022‐KY‐086) and Guangdong Provincial People's Hospital (#KY2024‐915‐02) of Southern Medical University, and the third People's Hospital of Zhongshan (#SSYLL20210301). All procedures performed in studies involving human participants were in accordance with the ethical standards of the institutional and/or national research committee and with the 1975 Helsinki declaration and its later amendments or comparable ethical standards. All participants in this study provided informed consent prior to the study following presentation of the nature of the procedures.

## Author Contributions

C.N., H.C., Q.C., and Y.L. contributed equally to this work. C.N., Z.W., F.Y., and C.Z. conceived and designed the experiments. C.N., H.C., Q.C., Y.L., Y.W., L.Y., X.W., H.N., S.L. performed the experiments. C.N., H.C., H.X., Z.W., F.Y., and C.Z. analyzed the data. T.J., Q.Y. collected and diagnosed the control and patient subjects. C.N., H.C., Z.W., F.Y., and C.Z. wrote the manuscript.

## Conflict of Interest

The authors declare no conflict of interest.

## Supporting information



Supporting Information

Supporting Information

## Data Availability

Raw data from RNA sequencing has been deposited in the GEO database under the accession code GSE302229 for murine brain tissues. All data supporting the findings described in this manuscript are available in the article and in the Supplementary Information and from the corresponding author upon reasonable request.
