# Supplementary material for: Allele‐Specific Regulation of PAXIP1‐AS1 by SMC3/CEBPB at rs112651172 in Psychiatric Disorders Drives Synaptic and Behavioral Dysfunctions in Mice
Source: Adv Sci (Weinh). 2025 Sep 5;12(44):e08259. doi: 10.1002/advs.202508259 (PMC12667460; doi:10.1002/advs.202508259)
Supplement: Supplementary file 1 — Supporting Information [file ADVS-12-e08259-s002.docx]

**Supplementary Figures**


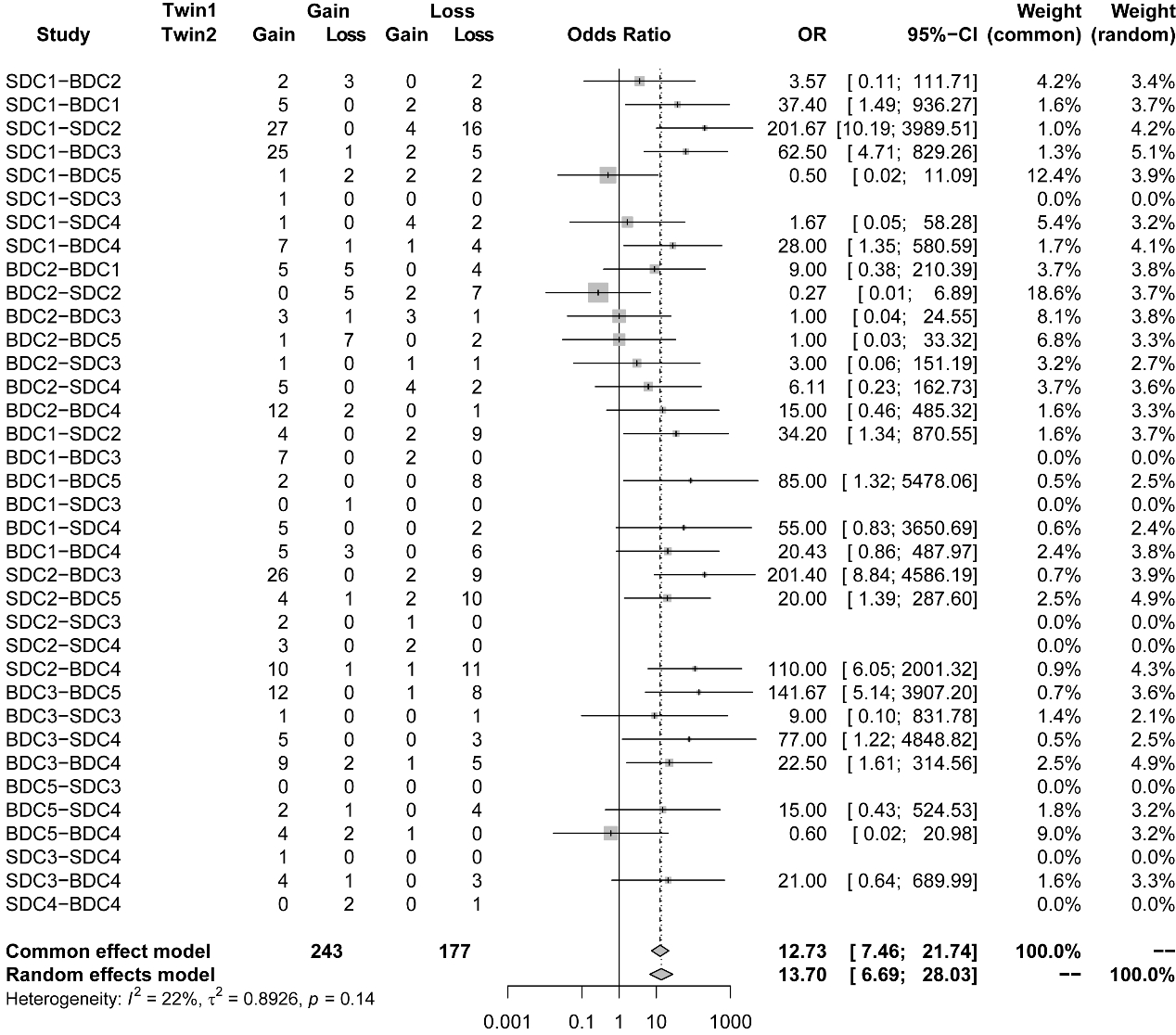


**Figure S1A**


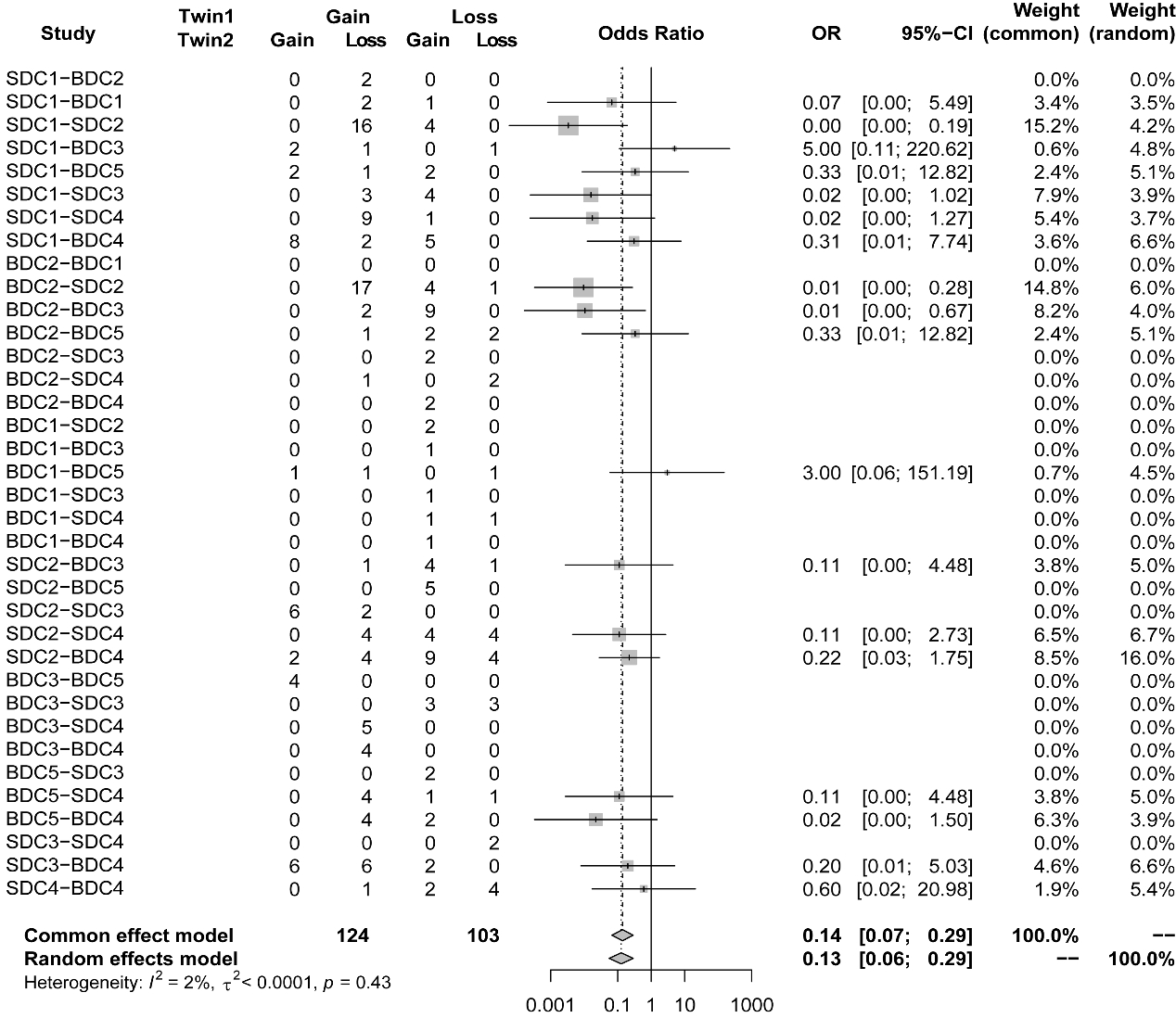


**Figure S1B**


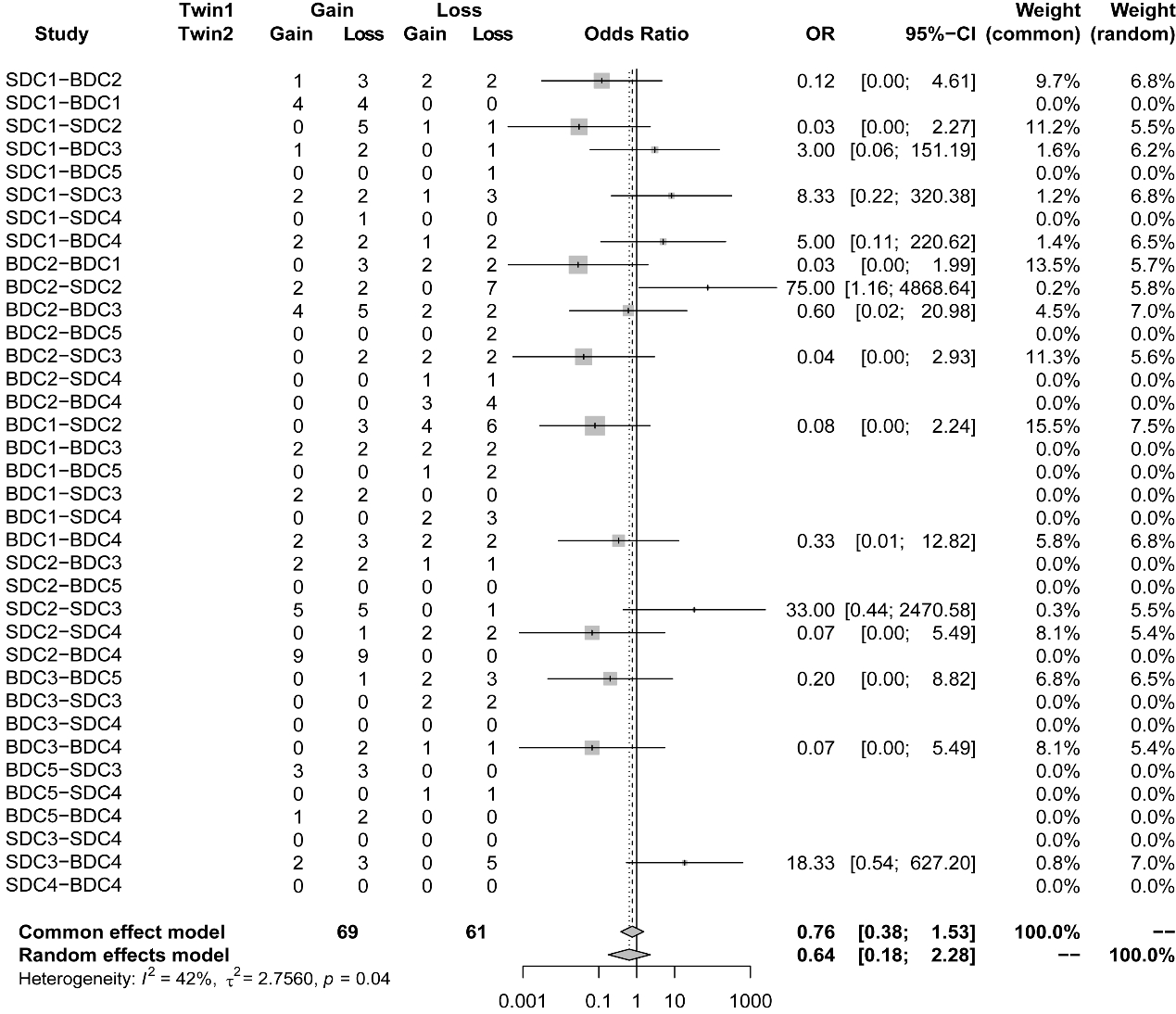


**Figure S1C**

**Figure S1** **Meta-analysis of ASE direction.** Meta-analysis of ASE direction by comparing ASE between any two PDC MZ twins across ASE lncRNA pairs, stratified by Bayes Factor (BF): high-confidence ASE pairs (BF>100; **A**), low-confidence pair (BF<0.01; **B**), and neutral pairs (1/3<BF<3; **C)**. "Gain" denotes a higher proportion of the Alt allele in the affected individual compared to their unaffected co-twin, while "Loss" indicates a lower proportion. "Gain-Gain" refers to twin pairs in which both individuals consistently show a Gain pattern. "Gain-Loss" and "Loss-Gain" represent discordant ASE directions between twins, with one twin showing Gain and the other Loss. "Loss-Loss" indicates consistent Loss across both twins. An odds ratio (OR) > 1 suggests concordant ASE directionality between twins, while OR < 1 indicates discordance in ASE transitions.


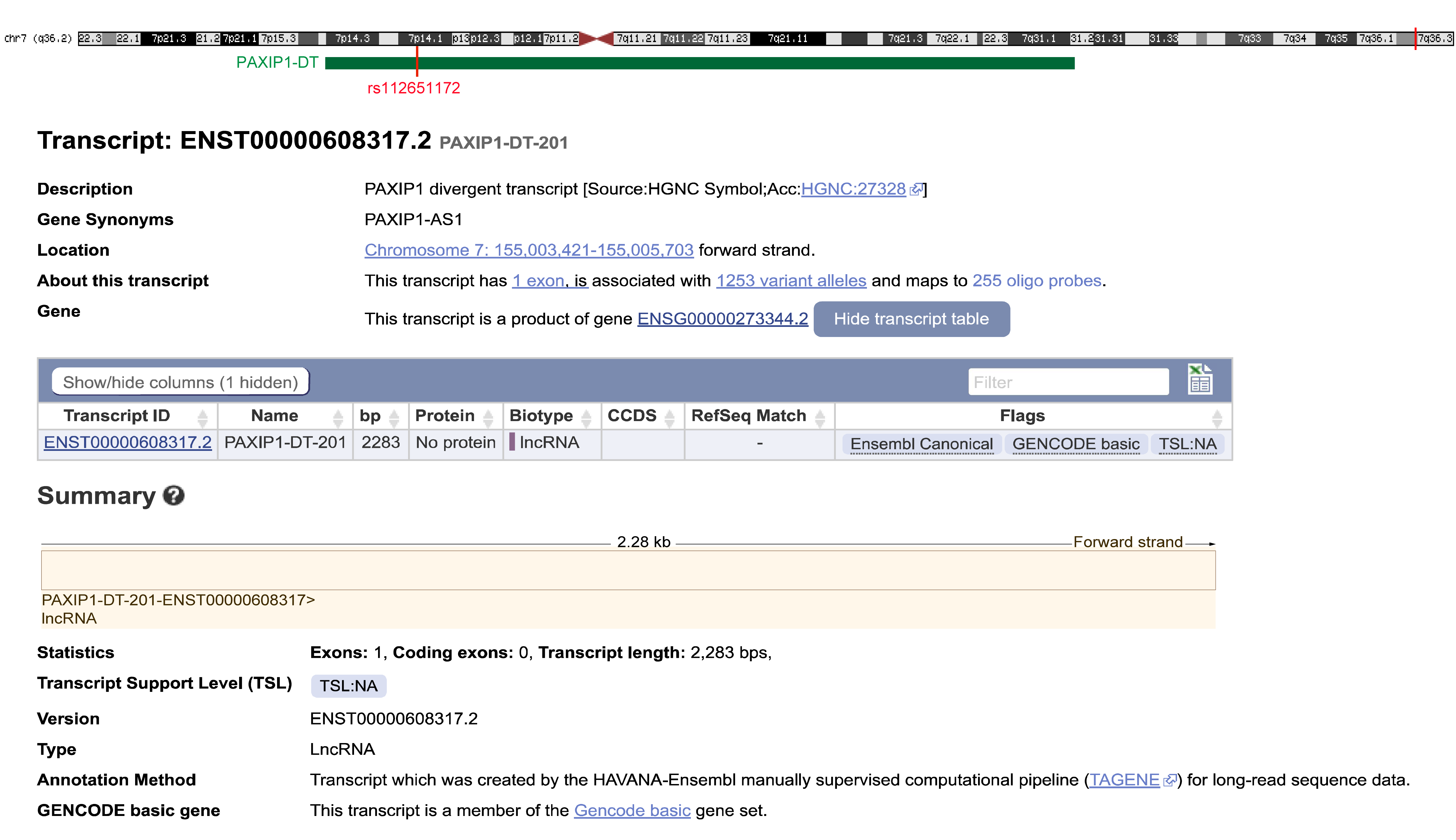


**Figure S2 Transcript information for *PAXIP1-AS1* retrieved from the Ensemble database.**

**
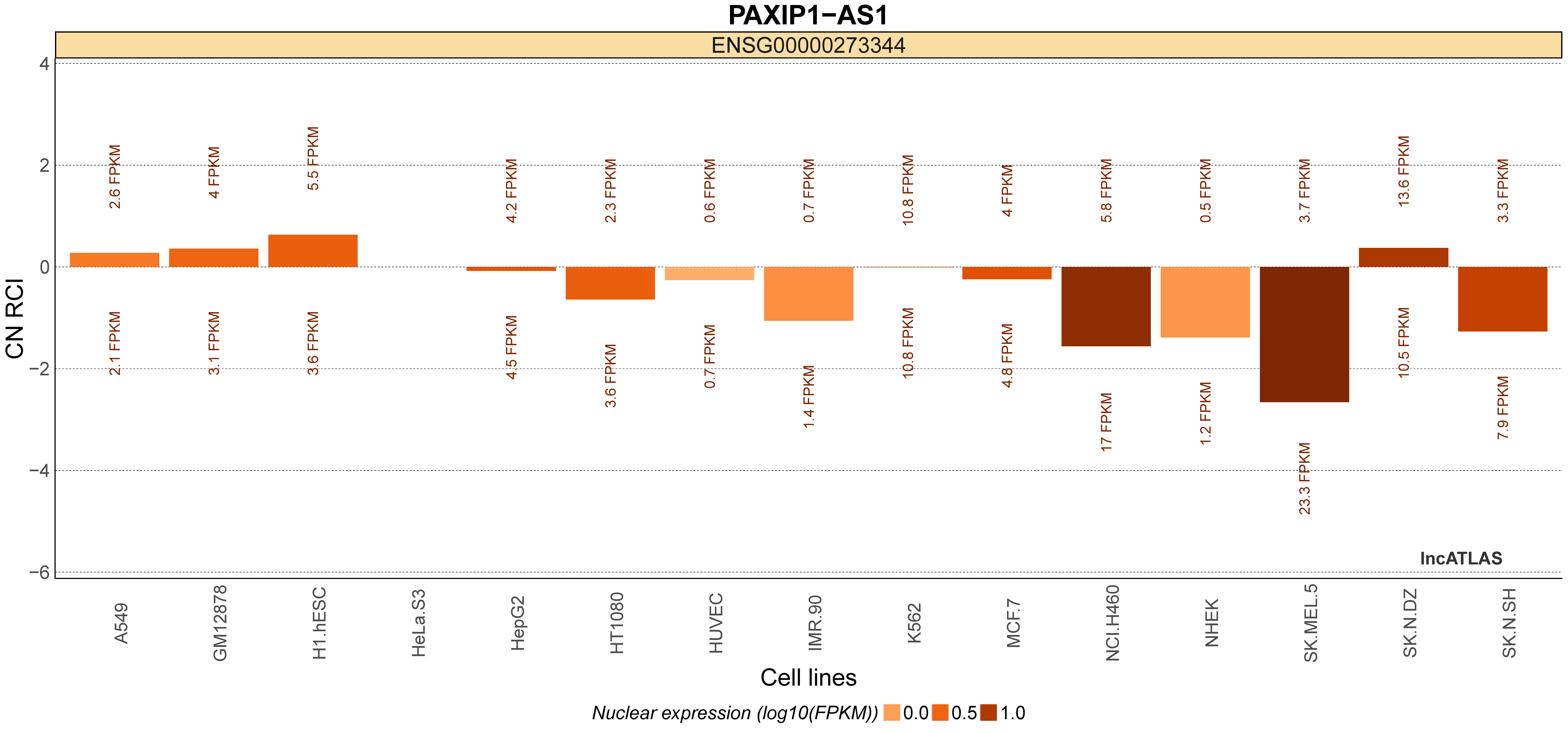
**

**Figure S3 Subcellular localization of *PAXIP1-AS1* across various cell lines, as reported in the lncATLAS database.** Data illustrate the relative enrichment of *PAXIP1-AS1* in nuclear versus cytoplasmic compartments, providing insights into its potential regulatory functions.

**Figure S4 Overexpression of different *PAXIP1-AS1* alleles (rs112651172 C/G) in the mouse prefrontal cortex and subsequent behavioral assessments. (A)** Recombinant adeno-associated virus (rAAV) vectors were stereotactically injected into the prefrontal cortex (PFC) of C57BL/6 wild-type mice to overexpress *PAXIP1-AS1*. Three groups were included: control AAV (Ctr), AAV expressing the *PAXIP1-AS1* transcript with the reference C allele (Ref), and AAV expressing the transcript with the alternative G allele (Alt). Top: fluorescence microscopy showing viral expression in PFC brain slices. Bottom: quantitative PCR (qPCR) confirming transgene expression in PFC brain tissues of Ctr (n=5), Ref (n=6) and Alt (n=6) mice **(B)** Experimental timeline (D, day) and flowchart of the behavioral testing battery performed 28 days post-injection.

**Figure S5 Allele-specific effects of *PAXIP1-AS1* overexpression on additional behavior and cognition measure in mice. (A, B)** Open field test (OFT): Total distance traveled and time spent in the center from Ctr (n=7), Ref (n=7) and Alt (n=9) female mice (**A**) and Ctr (n=10), Ref (n=10) and Alt (n=10) male mice (**B**), indicating general activity. **(C, D)** Y-maze: Total number of arm entries in Ctr (n=7), Ref (n=7) and Alt (n=9) female mice **(C)** and Ctr (n=10), Ref (n=10) and Alt (n=10) male mice (**D**), indicative of working memory. **(E, F)** Elevated plus maze (EPM): Time spent in open arms and number from Ctr (n=7), Ref (n=7) and Alt (n=9) female mice **(E)** and Ctr (n=10), Ref (n=10) and Alt (n=10) male mice (**F**), assessing anxiety-like behavior. **(G, H)** Barnes Maze: Escape latency across training days in Ctr (n=7), Ref (n=7) and Alt (n=9) female mice **(G)** and Ctr (n=10), Ref (n=10) and Alt (n=10) male mice (**H**), assessing spatial learning and memory. Data presented as mean ± SD for control (Ctr), *PAXIP1-AS1* reference allele (Ref), and *PAXIP1-AS1* alternative allele (Alt). Adjusted P values were calculated using ANOVA followed by Tukey’s multiple comparison test for the indicated comparisons. P < 0.05 was considered statistically significant (ns, not significant).


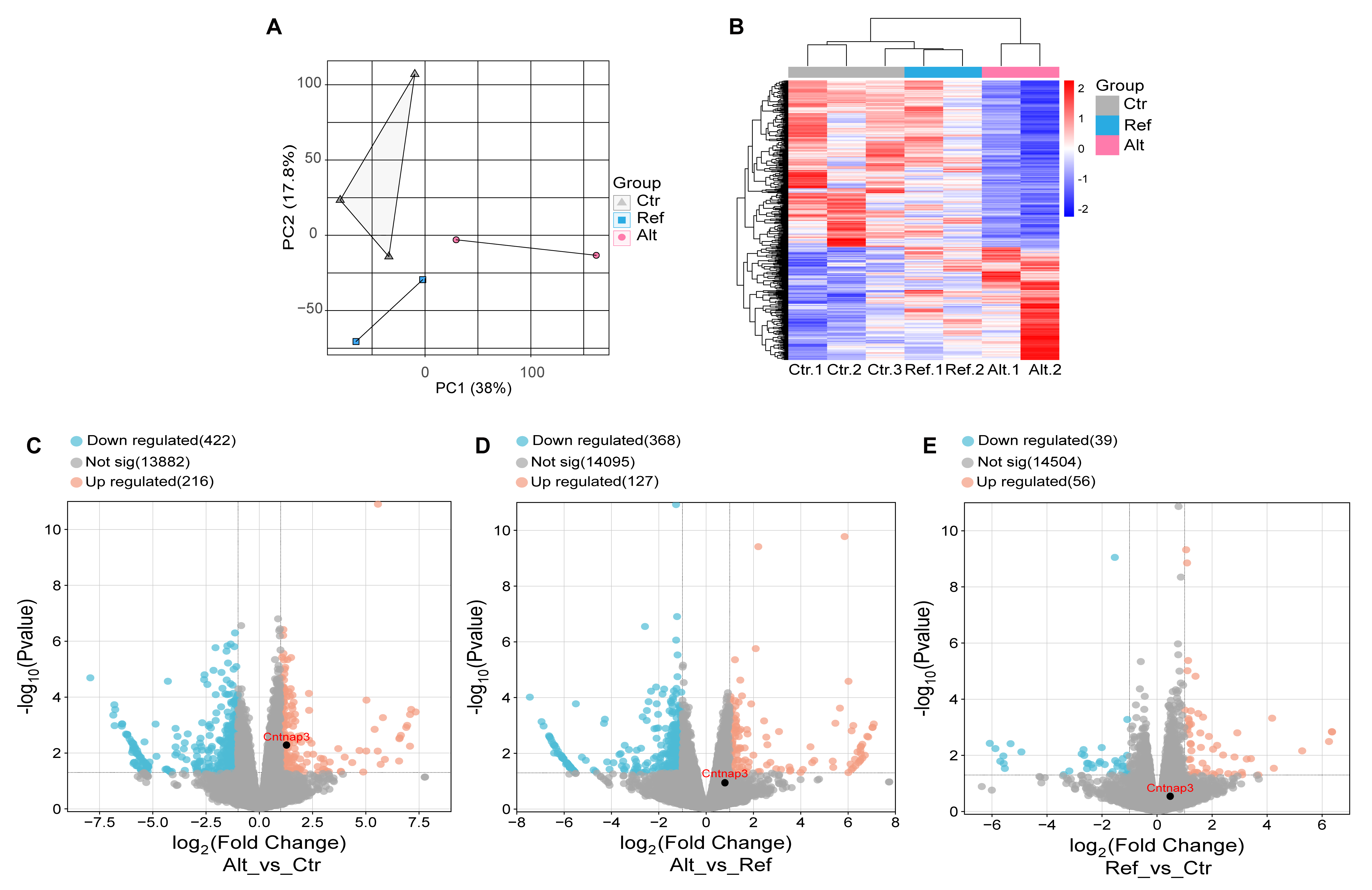


**Figure S6 RNA sequencing analysis of PFC tissue from Alt, Ref and Ctr mice. (A)** Principal component analysis (PCA) of transcriptome-wide gene expression data, illustrating sample variability and group clustering. **(B)** Hierarchical clustering heatmap based on gene expression profiles, showing the similarity and divergence among Alt, Ref, and Ctr samples. **(C-E)** Volcano plots depicting differential gene expression between groups. Colored dots represent significantly upregulated or downregulated genes based on fold change and statistical thresholds.


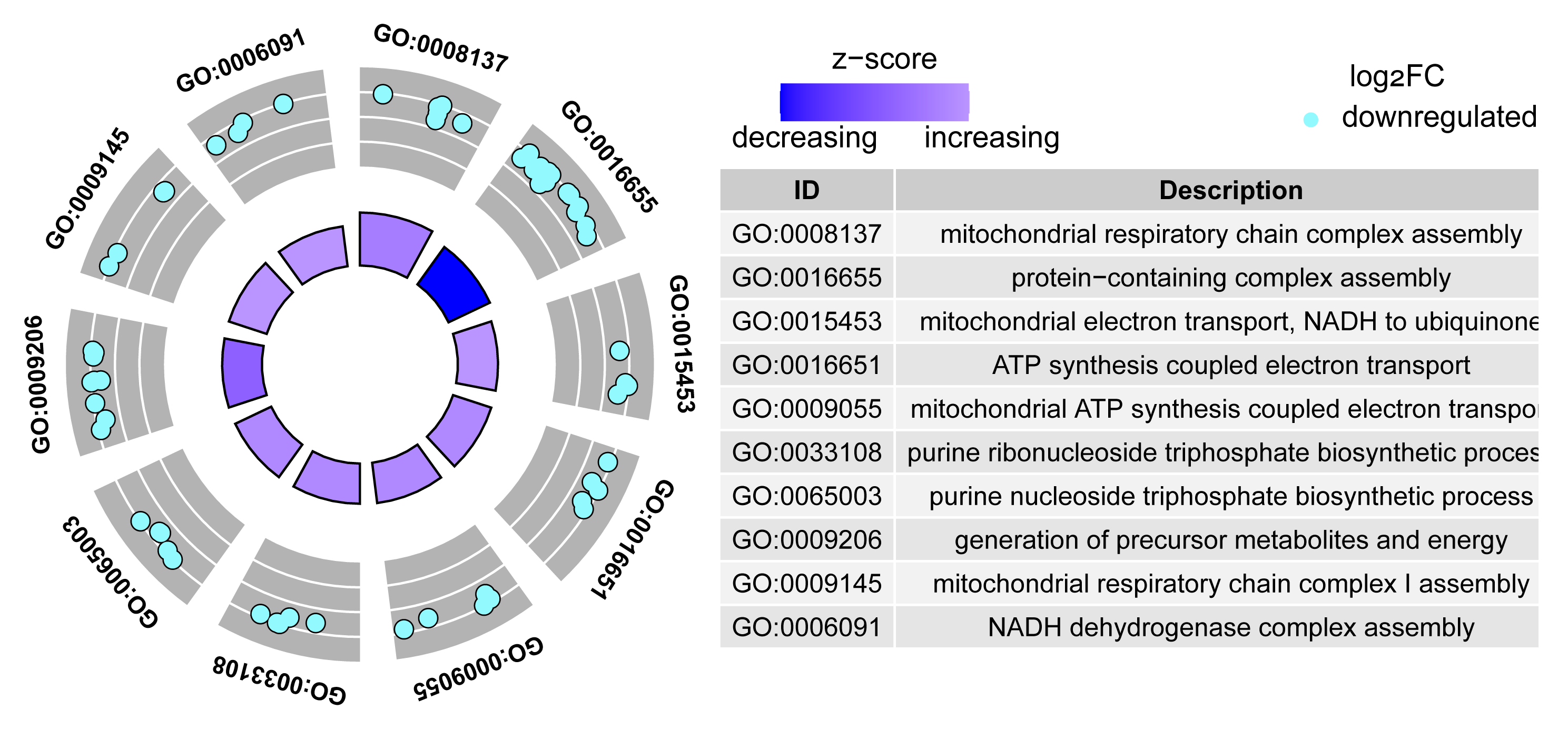


**Figure S7 Gene Ontology Biological Process (GO-BP) enrichment analysis of downregulated differentially expressed genes (DEGs) identified by DESeq2.** Analyses were performed using ordered categorical comparisons across the three experimental groups (Ctr, Ref, and Alt). Gene Ontology-Biological Process (GO-BP) enrichment analysis was performed using ToppGene Suite.


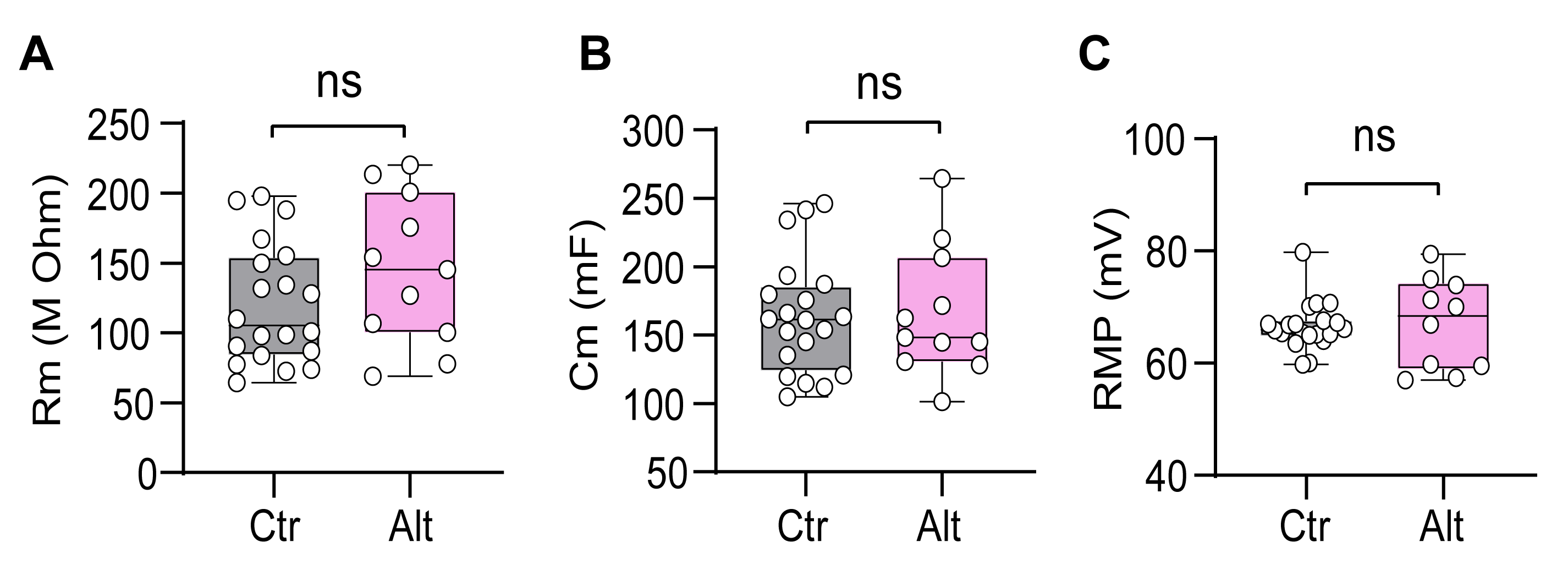


**Figure S8 Electrophysiological measurements in *PAXIP1-AS1* Alt overexpressing mice. (A-C)** Membrane resistance recorded from layer 5 cortical neurons of Ctr mice (20 neurons) and Alt mice (11 neurons) **(A)**, membrane capacitance recorded from Ctr mice (20 neurons) and Alt mice (11 neurons) **(B)** and resting membrane potential recorded from Ctr mice (20 neurons) and Alt mice (10 neurons) **(C)**. Data are presented as mean ± SD for cells obtained from at least three mice per group (Ctr and Alt). P values were calculated using unpaired two-tailed Student’s t-tests. P < 0.05 was considered statistically significant (ns, not significant).


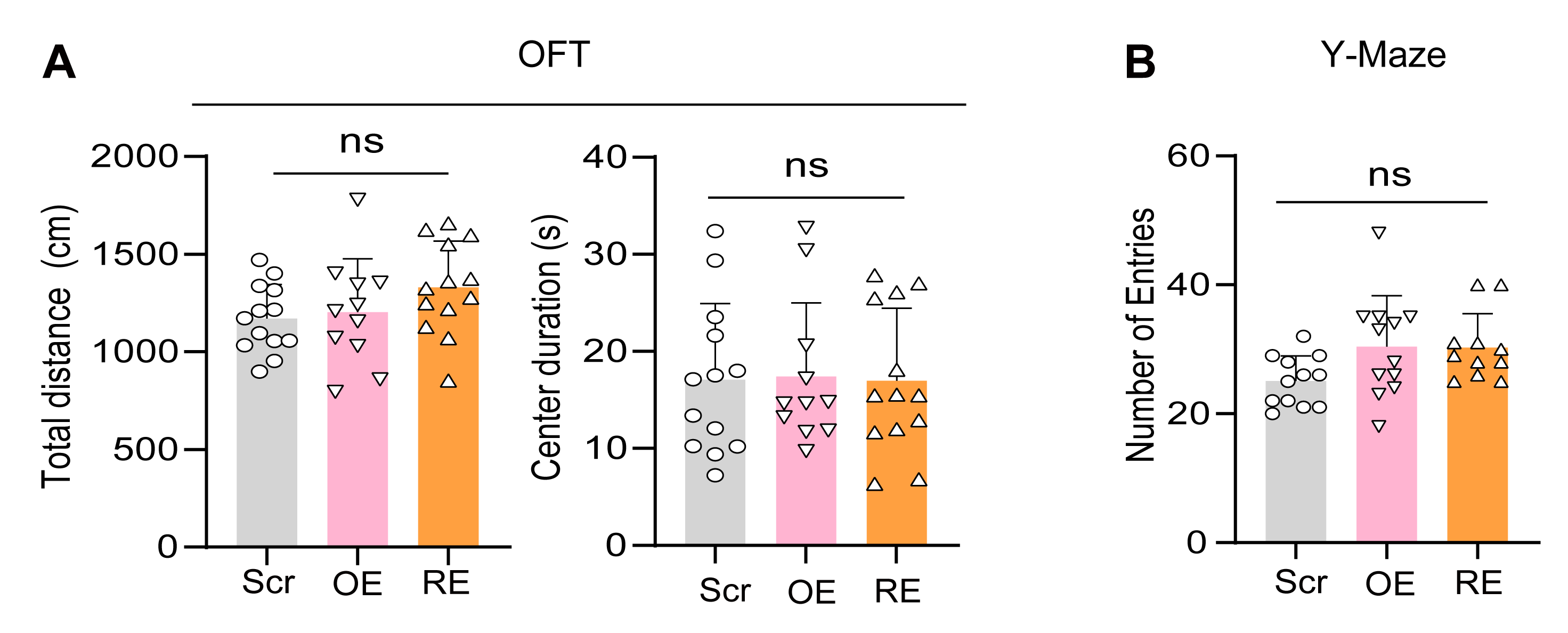


**Figure S9 Behavioral assessment of recovered *CNTNAP3* expression in *PAXIP1-AS1*-overexpressing female mice. (A)** Total distance traveled and center dwell time in the Open Field Test (OFT) from Ctr (n=13), Ref (n=11) and Alt (n=13) female mice, and **(B)** Number of entries in the Y-maze test from Ctr (n=12), Ref (n=12) and Alt (n=11) female mice. Data are presented as mean ± SD for Scr (gray bars), OE (pink bars), and RE (orange bars) groups, with at least eleven mice per group. Adjusted P values were calculated using ANOVA followed by Tukey’s multiple comparison test for the indicated comparisons. P < 0.05 was considered statistically significant (ns, not significant).


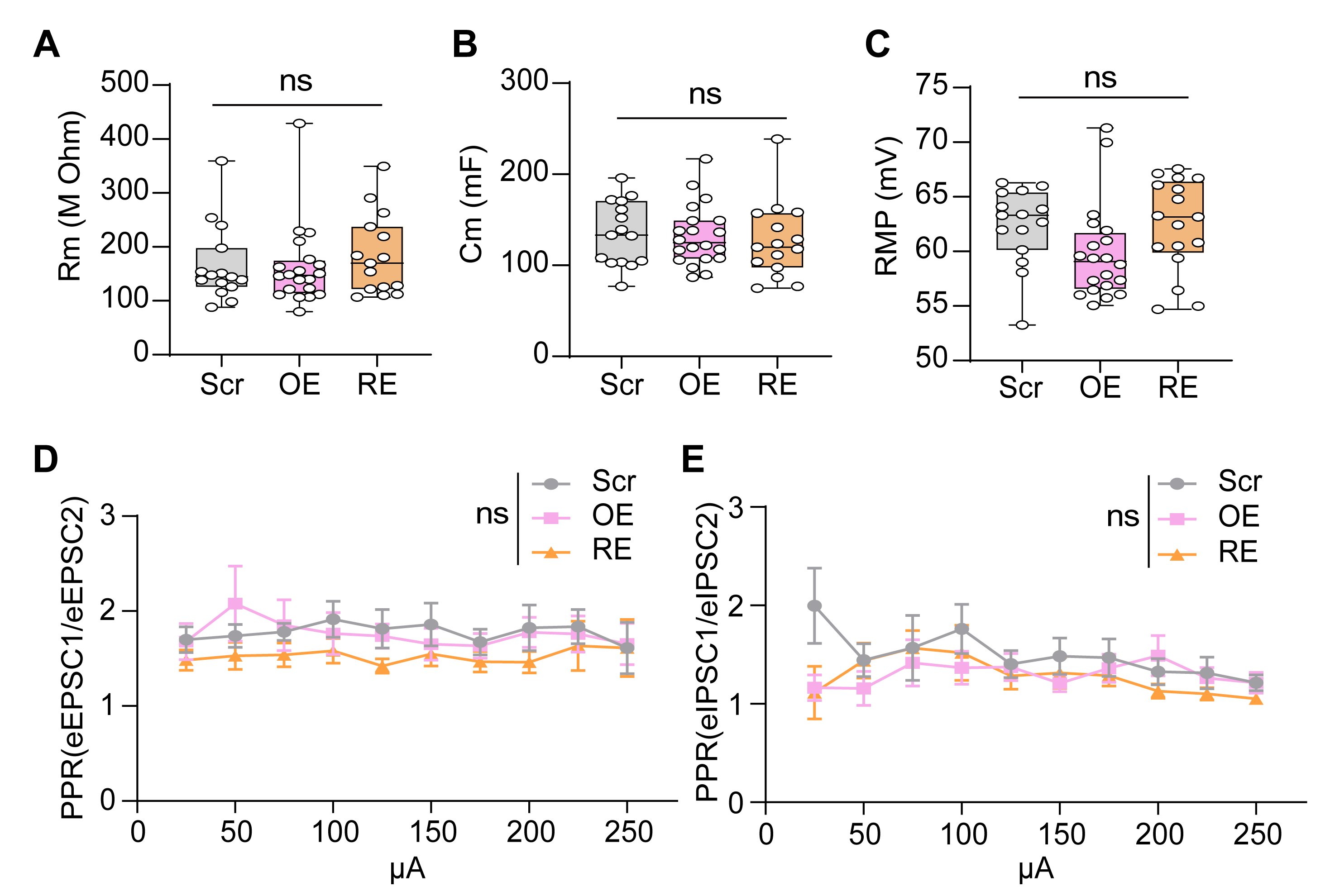


**Figure S10 Electrophysiological assessment of recovered *CNTNAP3* expression in *PAXIP1-AS1*-overexpressing mice. (A)** Membrane resistance recorded from layer 5 cortical neurons of Scr mice (15 neurons), OE mice (20 neurons) and RE mice (15 neurons), **(B)** membrane capacitance recorded from Scr mice (15 neurons), OE mice (20 neurons) and RE mice (15 neurons), **(C)** resting membrane potential recorded from Scr mice (15 neurons), OE mice (20 neurons) and RE mice (17 neurons), and **(D)** PPRs of eEPSCs in layer 5 cortical neurons from Scr mice (10 neurons), OE mice (14 neurons) and RE mice (13 neurons) and **(E)** eIPSCs plotted against interstimulus intervals from Scr mice (10 neurons), OE mice (13 neurons) and RE mice (12 neurons). Data are presented as mean ± SD. P values were calculated using ANOVA followed by followed by Tukey’s multiple comparison test for the indicated comparisons. P < 0.05 was considered statistically significant (ns, not significant).
